# Hospital acquisitions and 30-day mortality after acute myocardial infarction and stroke in Germany: a quasi-experimental cohort study

**DOI:** 10.1016/j.lanepe.2026.101788

**Published:** 2026-07-23

**Authors:** Esra Eren Bayindir, Reinhard Busse, Jonas Schreyögg

**Affiliations:** a Hamburg Center for Health Economics, University of Hamburg, Hamburg, Germany; b Department of Health Care Management, Berlin University of Technology, Berlin, Germany

**Keywords:** Hospital acquisitions, Quality of care, Acute myocardial infarction, Stroke, Germany

## Abstract

**Background:**

Hospital consolidation has intensified globally, but evidence regarding its association with clinical quality remains mixed, particularly in universal healthcare systems with regulated prices. We assessed whether hospital acquisitions were associated with changes in 30-day excess mortality for acute myocardial infarction (AMI) and stroke.

**Methods:**

This quasi-experimental cohort study used a staggered difference-in-differences design analyzing German hospitals providing AMI or stroke care (2009–2019). We compared performance changes three years pre- and post-acquisition between acquired hospitals (n = 125 for AMI; n = 121 for stroke) and control hospitals with stable ownership (n = 821 for AMI; n = 830 for stroke). Primary outcomes were 30-day excess mortality rates. Secondary outcomes included medical staffing intensity and the provision of cardiac catheterization laboratories and stroke units.

**Findings:**

Acquisition was associated with a 1.24 percentage-point (pp) decline in excess 30-day AMI mortality within three years (95% CI −2.04 to −0.44; p = 0.002). This improvement coincided with a 6.63 pp increase in the probability of providing cardiac catheterization laboratories (0.75 to 12.52; p = 0.03). For stroke, the association with excess 30-day mortality indicated a clinically relevant but statistically insignificant 0.43 pp decline (−1.38 to 0.53; p = 0.44). Nurse staffing intensity transiently increased during the acquisition year for both AMI and stroke.

**Interpretation:**

In a regulated, fixed-price system, acquisitions were significantly associated with reduced AMI mortality and expanded interventional infrastructure. Findings suggest consolidation can improve clinical quality by unlocking capital for life-saving investments. While capital-intensive conditions like AMI benefit rapidly, complex care pathways like stroke likely require additional clinical integration strategies.

**Funding:**

German Research Foundation.

**Research in context**

**Evidence before this study**

We systematically searched PubMed and Google Scholar from database inception to January 31, 2026, for studies on the impact of hospital mergers and acquisitions on quality of care, using the terms “hospital” AND (“merger” OR “acquisition”) AND “quality” without publication date restrictions. Hospital consolidation has accelerated globally, prompting intense debate among regulators regarding its impact on patient care. However, existing empirical evidence is predominantly derived from the United States—a mixed-payer system where consolidation often leads to increased prices, with mixed or detrimental effects on clinical quality. Evidence remains scarce regarding the impact of consolidation in universal healthcare systems with fixed-price reimbursement models, such as the Diagnosis-Related Groups (DRGs) used in Germany and across much of Europe. In these regulated environments, financially distressed hospitals cannot raise prices to overcome deficits. Consequently, there is a critical lack of robust, quasi-experimental evidence examining whether consolidation under these conditions might actually improve quality of care by unlocking the capital necessary for vital clinical infrastructure.

**Added value of this study**

To our knowledge, this is the first study to provide national, quasi-experimental evidence on the effect of hospital acquisitions on the quality of emergency care (acute myocardial infarction [AMI] and stroke) in a purely fixed-price, universal healthcare system. Using a rigorous staggered difference-in-differences design on data from 1020 German hospitals (2009–2019), we demonstrate that hospital acquisitions were associated with a significant 1.24 percentage-point reduction in excess 30-day mortality for AMI. Crucially, we identify a primary mechanism for this improvement: acquisitions enabled capital-intensive infrastructural investments, evidenced by a 6.63 percentage-point increase in the probability of operating a cardiac catheterization laboratory, alongside transient increases in medical staffing. For stroke, while average mortality did not significantly change, cohort-specific analyses revealed quality improvements in later acquisition years. Furthermore, our stratification analysis demonstrates that these clinical improvements occurred even in highly concentrated local hospital markets, where competitive pressure is lowest.

**Implications of all the available evidence**

In regulated healthcare systems where reimbursement rates are fixed, struggling hospitals cannot raise prices to overcome financial deficits or invest in clinical quality. Our findings suggest that, in such environments, hospital consolidation can serve as a vital mechanism to overcome financial distress and secure capital for life-saving infrastructure. For health policymakers and regulators, these results indicate that allowing mergers can significantly improve clinical outcomes for capital-intensive, standardized emergency conditions like AMI. However, achieving universal quality gains across all service lines—particularly for complex, labor-intensive pathways like stroke care—likely requires targeted clinical integration strategies that go beyond infrastructural investment.

## Introduction

Hospital acquisitions—hereafter used to refer broadly to all verified changes in ownership, including mergers and acquisitions—have dramatically reshaped the healthcare landscape globally over the past two decades.^1^, ^2^, ^3^, ^4^, ^5^, ^6^, ^7^ In the United States, extensive empirical evidence shows that hospital consolidation leads to increased negotiated prices for privately insured patients,^8^^,^^9^ raising significant concerns about patient welfare. However, the associated effects on the quality of patient care remain a subject of active debate and mixed empirical findings.^10^^,^^11^ Prior studies examining the US market generally report either modest deterioration in patient experiences or no significant changes in health outcomes following acquisitions,^12^, ^13^, ^14^, ^15^ challenging the argument that consolidation-driven efficiency gains translate into widespread quality improvement.^16^

Crucially, the vast majority of this literature focuses on health care systems where market power allows for price increases. Less is known about the consequences of acquisitions in non-market-driven settings.^17^ In universal healthcare systems with fixed-price regulatory environments, the primary pathway for economic benefit from consolidation is through providing high-tech services and operational efficiencies (e.g., lower administrative costs, bulk purchasing) rather than increased market power and pricing leverage.^13^ This institutional context fundamentally alters the incentives for acquired hospitals: without the ability to raise prices, one would expect hospitals to rely on provision of more advanced treatment options and standardization of clinical processes, which may improve health outcomes.^2^^,^^9^^,^^18^ This organizational and infrastructural focus is a recognized determinant of survival in acute cardiovascular emergencies.^19^ Alternatively, acquiring systems might pursue aggressive cost-cutting measures to achieve financial viability, potentially leading to reduced staffing ratios or the complete closure of complex, resource-intensive service lines.^10^, ^11^, ^12^

Theoretical literature generally predicts that in fixed-price markets, reduced competition leads to lower quality.^9^ However, as Gaynor et al. note, this prediction relies on the assumption that the regulated price is set above marginal cost.^9^ In health systems where regulated prices may fall below marginal cost for some providers—a “negative margin” scenario described by Gowrisankaran and Town (2003)—this prediction reverses.^20^ In such settings, intense competition can degrade quality due to financial strain, whereas consolidation may unlock the economies of scale necessary to invest in quality-improving infrastructure. In this context, consolidation offers a pathway to financial stability by possibly spreading high fixed costs over a larger patient base and improving access to capital markets. These financial efficiencies and centralized capital reserves can enable the rapid acquisition of expensive medical technologies—such as cardiac catheterization laboratories—that standalone hospitals, particularly those facing insolvencies, might otherwise be unable to afford.

This distinction is critical for the German context, where Diagnosis Related Group (DRG) reimbursement rates are calculated based on average costs, effectively setting prices below marginal cost for a subset of hospitals.^21^ Recent empirical evidence supports this “negative margin” mechanism; for example, higher competition in England has been linked to lower quality for hip and knee replacements,^22^ and increased competition for AMI care in Germany was found to yield no quality benefits.^18^

In this quasi-experimental cohort study, we exploit the hospital acquisitions that took place between 2012 and 2016 within a universal health care system characterized by fixed prices. We employ a dynamic difference-in-differences event study approach with staggered adoption to examine the effect of acquisition on hard measures of quality: excess 30-day mortality rates for two high-volume emergency conditions, acute myocardial infarction (AMI) and stroke. Furthermore, we investigate potential mechanisms, specifically focusing on changes in availability of cardiac catheterization laboratories, stroke units, and clinical staffing intensity (nurses and medical doctors per patient day), which may mediate changes in quality.

## Methods

### Study population

**Fig. 1:**
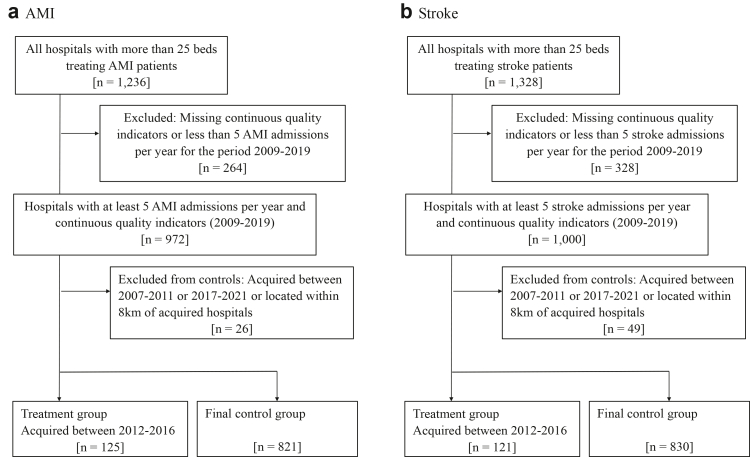
Flow diagram of the hospital selection process. The flowchart details the inclusion and exclusion criteria applied to identify the final samples of acquired and control hospitals for the (a) acute myocardial infarction (AMI) and (b) stroke analyses.

For this quasi-experimental cohort study, we identified hospital acquisitions by analyzing changes in reported hospital ownership in publicly available structured quality reports and hospital directory from 2007 to 2021. To ensure valid identification of ownership changes and exclude corporate restructuring or simple name changes, we validated every potential transaction through manual web searches, reviewing hospital press releases, local news archives, and corporate history pages. Only verified transfers of ownership between distinct entities were included in the treatment group. The detailed hospital selection and exclusion process for both clinical cohorts is illustrated in Fig. 1.

We used quality indicators between 2009 and 2019 to examine the impact of hospital acquisitions that were realized between 2012 and 2016. This window ensures all acquired hospitals could be consistently followed for three full years pre- and post-acquisition.

To create a clean control group, we utilized hospital acquisition data from the entire 2007–2021 period to exclude any hospitals that experienced a change in ownership outside the core 2012–2016 treatment window. Finally, the analysis was strictly limited to the pre-pandemic period to eliminate potential confounding effects related to the COVID-19 pandemic.

Our study sample initially included all short-term acute care hospitals with at least 25 beds treating AMI (n = 1236) or stroke (n = 1328) patients. From this initial pool, we required hospitals to have a minimum of five condition-specific admissions in each year. Hospitals with any missing performance data were excluded to maintain consistent inclusion criteria for both acquired and control hospitals throughout the study period.

The treatment group was defined as all hospitals that underwent a change in ownership, during the period of 2012–2016. This window allowed for the analysis of performance changes over three years post-transaction. Following the application of all exclusion criteria, this resulted in a final treatment group of 125 hospitals for the AMI analysis and 121 hospitals for the stroke analysis.

The control group comprised all other hospitals that met the initial inclusion criteria and were subject to two strict exclusions. First, they were not acquired throughout the entire 2007–2021 period. Second, for our primary analysis, we excluded potential local competitors, defined as hospitals located within an eight km (five miles) radius of any acquired hospital, following Beaulieu et al. (2020).^15^ This exclusion was implemented to reduce potential bias from spillover effects. Applying these criteria yielded a final control group of 821 hospitals for the AMI analysis and 830 hospitals for the stroke analysis. Additionally, to ensure our findings were robust to broader geographic market definitions, we also tested a larger 15 km exclusion radius in our sensitivity analyses.

### Outcome measures

The primary outcomes were the excess 30-day mortality rates for AMI and stroke admissions. Excess mortality is calculated as the difference between the observed and the expected 30-day mortality rate, using a time-consistent risk-adjustment model (details on outcome measures are in Appendix A).

Secondary outcomes included secondary clinical measures—specifically, the raw 30-day mortality rates and 30-day readmission rates for both AMI and stroke—as well as structural and resource mechanisms. To explore potential mechanisms driving our primary findings, these secondary structural outcomes included changes in patient volume, medical staffing intensity (the number of medical doctors and nurses per patient day), and the provision of cardiac catheterization laboratories and stroke units (details on the construction of these variables are in Appendix B). We identified these units using specific Operation and Procedure Statistics codes from the structured quality reports.^23^ To rule out coding errors and ensure the units were operational, we defined a hospital as having a unit only if at least three such procedures were documented in a given year.

### Covariates and entropy balancing

To perform entropy balancing, we used the number of hospital beds, patient volume for AMI and stroke, and an exogenous measure of hospital competition at baseline (described in Appendix C). We also included baseline hospital bed utilization rate as a proxy for financial performance, as capacity utilization is a primary determinant of insolvency risk in the German DRG system.^24^

### Statistical methods

Our unit of observation was the hospital-year, utilizing a balanced panel dataset structure where each row represented a single hospital in a specific year from 2009 to 2019. We estimated the association between hospital acquisitions and quality using the Callaway and Sant’Anna (2021) difference-in-differences estimator,^25^ which is robust to heterogeneous treatment effects in staggered adoption designs. This method estimates the group-time average treatment effect on the treated, controlling for time-invariant hospital confounders.

All observations were weighted according to the baseline number of relevant admissions (i.e., AMI and stroke admissions) at each hospital. By restricting our primary analysis to emergency cases (AMI and stroke), we aimed to mitigate potential patient selection and changes in patient composition that might result from the hospital acquisition.

The core identification assumption of the difference-in-differences analysis is the plausibility of parallel trends, which posits that, absent the acquisition, the quality of care trends for the acquired and control hospitals would have remained parallel in the post-transaction period. We formally assessed this assumption by comparing the changes in outcomes between acquired and control hospitals during the pre-transaction period.

Given that hospital acquisitions are non-random and potentially endogenous, we performed a secondary analysis employing entropy balancing to explicitly adjust the control group to be statistically equivalent to the acquired group based on observed baseline characteristics.

To examine heterogeneity, we stratified hospitals by baseline market competition—measured using a predicted Herfindahl-Hirschman Index (HHI) calculated following Gowrisankaran and Town (2003)^20^—to test the theoretical prediction regarding market power. We also stratified by bed utilization rates to examine heterogeneity by financial performance and evaluated temporal heterogeneity by acquisition cohort. Furthermore, to address underlying structural variations within the control cohort, we conducted a sensitivity analysis that subdivided the control group into individual, independent hospitals and system-affiliated hospitals.

To elucidate potential mechanisms, we applied the difference-in-differences estimator to our secondary outcomes: medical staffing intensity, patient volume, and the probability of operating cardiac catheterization laboratories or stroke units. To explicitly evaluate the competing hypothesis of cost-cutting through service reduction, we also evaluated the probability of service line interruption, defined as the cessation of AMI or stroke quality indicator reporting among hospitals providing these respective services at baseline. Finally, to estimate potential spillover effects, we substituted local competitors (within 8-km and 15-km radii) for the acquired hospitals in the estimation.

Primary outcomes were pre-specified to mitigate the risks associated with multiple testing; findings for all other outcomes are intended to be exploratory and should be interpreted in the context of their consistency with the primary clinical results. All statistical analyses were conducted in StataNow 19 MP. The study followed the Strengthening the Reporting of Observational studies in Epidemiology (STROBE) checklist.

### Ethics approval

The study was conducted in accordance with the Declaration of Helsinki and approved by the Institutional Review Board of University of Hamburg Business School on 15.09.2024 (the committee does not issue specific approval numbers). Because this study relied exclusively on the retrospective analysis of hospital-level, risk-adjusted quality indicators provided by the statutory health insurance system, the requirement for individual patient informed consent was waived by the ethics committee.

### Role of the funding source

The funder of the study had no role in study design, data collection, data analysis, data interpretation, or writing of the report.

## Results

For the AMI analysis, our sample included 125 acquired and 821 control hospitals (treating 710,584 cases), and for the stroke analysis, it included 121 acquired and 830 control hospitals (treating 955,878 cases) between 2009 and 2019. The unadjusted 30-day mortality rates in the dataset were 12.82% for AMI and 13.53% for stroke. At baseline, compared to the control group, acquired hospitals tended to have fewer beds, were less frequently publicly owned, more frequently not-for-profit or for-profit, and were less likely to have a stroke unit, though they were similar across other measured characteristics (Table 1). To provide broader visual context for our subsequent event study estimates, we plotted the annual, unadjusted trends of our primary and secondary health outcomes for both the acquired and control groups over the entire study period (2009–2019) (Supplementary Figure S1).

**Table 1:** Hospital characteristics for control and acquired hospitals at baseline.

Characteristics	Control hospitals	Acquired hospitals	Difference (95% CI)
	(N = 886)	(N = 134)	
Mean number of beds	404.78	328.73	76.05 (32.39–119.72)
For profit (%)	19.81	40.77	−20.96 (−29.93 to −11.99)
Not for profit (%)	39.86	46.92	−7.06 (−16.36 to −2.22)
Public (%)	40.33	12.31	28.02 (21.42–34.63)
Urban (%)	61.80	57.46	4.35 (−4.71 to 13.40)
Number of AMI cases	193.58	157.34	36.24 (−1.19 to 73.66)
Number of stroke cases	250.66	205.72	44.94 (−7.39 to 97.28)
MD intensity (AMI)	1.32	1.24	0.08 (−0.14 to 0.29)
Nurse intensity (AMI)	2.65	2.69	−0.04 (−0.66 to 0.58)
MD intensity (stroke)	1.34	1.41	−0.07 (−0.39 to 0.24)
Nurse intensity (stroke)	3.05	3.65	−0.60 (−1.75 to 0.55)
Cathlab (%)	49.20	44.03	5.18 (−3.94 to 14.30)
Stroke unit (%)	40.07	29.10	10.97 (2.54–19.39)
Predicted HHI, AMI	0.39	0.39	0.00 (−0.03 to 0.04)
Predicted HHI, stroke	0.35	0.36	−0.01 (−0.04 to 0.03)

CI stands for confidence interval. AMI stand for acute myocardial infarction. MD intensity and nurse intensity are number of medical doctors and nurses per 1000 patient day (explained in Appendix B). Cathlab stands for cardiac catheterization laboratory. HHI stands for Herfindahl Hirschman Index (explained in Appendix C). N represents the total unique hospitals pooled across both the AMI and stroke cohorts for baseline descriptive purposes.

We assessed the parallel trends assumption by conducting formal tests of the joint hypothesis that pre-transaction coefficients are zero (Supplementary Table S1). Trends for mortality, readmission, patient volume, and stroke unit availability were statistically indistinguishable between acquired and control hospitals. However, we observed evidence of differential pre-trends for medical doctor intensity for AMI (p = 0.03) and nurse intensity for stroke (p = 0.02), as well as a borderline result for cardiac catheterization laboratories (p = 0.06).

**Fig. 2:**
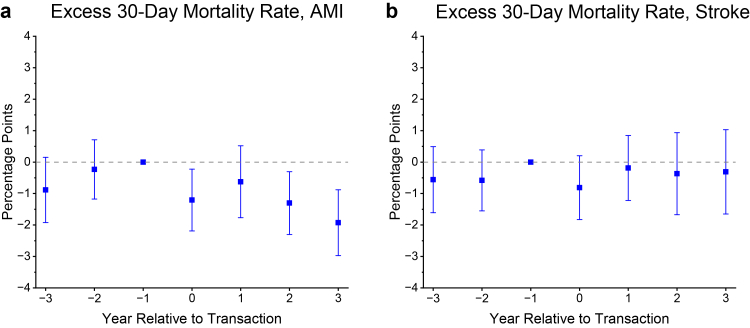
Primary outcomes: Dynamic event-study estimates of the association between hospital acquisition and excess 30-day mortality for acute myocardial infarction (AMI) and stroke. The panels display difference-in-differences estimates comparing acquired hospitals to control hospitals over a three-year pre- and post-acquisition period for (a) AMI and (b) stroke. The year immediately preceding the acquisition (Year −1) serves as the reference period. Data points represent the adjusted percentage-point difference in excess mortality. Error bars denote 95% confidence intervals.

**Fig. 3:**
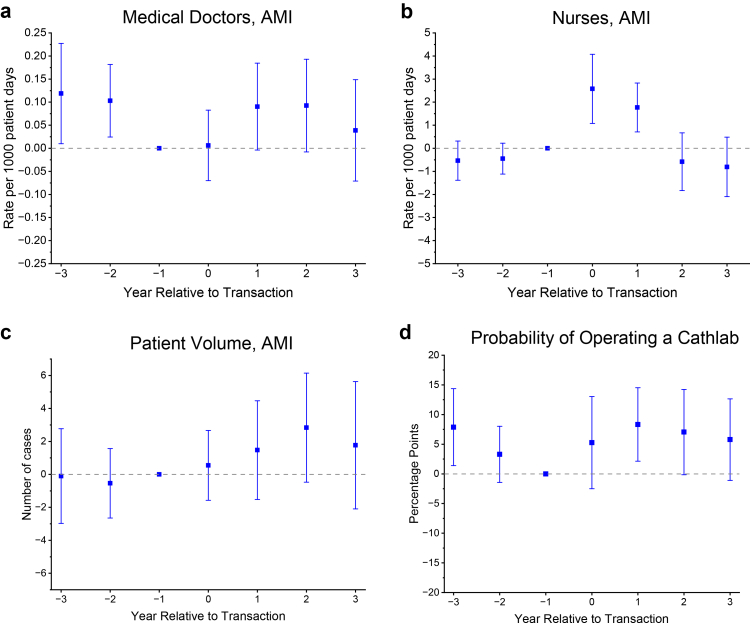
Secondary outcomes: Dynamic event-study estimates of the association between hospital acquisition and secondary outcomes for acute myocardial infarction (AMI). The panels illustrate difference-in-differences estimates for (a) medical doctor intensity, (b) nurse intensity, (c) AMI patient volume, and (d) the probability of operating a cardiac catheterization laboratory. Estimates compare acquired hospitals to independent control hospitals, with the year prior to acquisition (Year −1) serving as the reference period. Error bars denote 95% confidence intervals.

Regarding the primary outcome for AMI, acquisition was associated with a 1.24 percentage-point (pp) decline in excess 30-day AMI mortality over three years (95% confidence interval (CI) from −2.04 to −0.44, p = 0.002) (Table 2) Against a baseline mortality of 12.82%, this represents a 9.7% relative reduction in mortality. This effect strengthened to a 1.93 pp decline (95% CI −2.97 to −0.88, p < 0.001) by the third year (Fig. 2). This clinical improvement coincided with significant changes in secondary mechanistic outcomes: a 6.63 pp increase in the likelihood of operating a cardiac catheterization laboratory (95% CI from 0.75 to 12.52, p = 0.03) (Fig. 3). To address the concern that opening a catheterization laboratory might alter the severity mix of incoming patients, we compared our primary risk-adjusted results with unadjusted outcomes. Raw 30-day mortality rates tracked the risk-adjusted estimates closely (Supplementary Figure S2), suggesting that the observed improvements were not driven by unobserved changes in patient severity or selection bias. Secondary outcomes for staffing exhibited a transient increase, with nurse intensity rising significantly in the year of acquisition (2.58 per 1000 patient days, 95% CI from 1.08 to 4.08, p = 0.001) and the subsequent year (1.77 per 1000 patient days; 95% CI from 0.71 to 2.83, p = 0.001), before returning to baseline levels in the second- and third-years post-acquisition.

**Table 2:** Difference-in-differences estimates of hospital acquisition on clinical outcomes, staffing, and service availability for acute myocardial infarction and stroke.

Outcomes	DiD estimate (95% CI)	
	AMI	Stroke
Primary outcome		
Excess 30-day mortality	−0.012 (−0.020 to −0.004)	−0.004 (−0.014 to 0.005)
Secondary outcomes		
Raw 30-day mortality	−0.009 (−0.019 to 0.000)	−0.007 (−0.017 to 0.004)
30-day readmission rate	0.001 (−0.011 to 0.012)	−0.001 (−0.008 to 0.007)
Staffing and volume		
MD intensity (per 1000 PD)	0.06 (−0.02 to 0.13)	−0.05 (−0.13 to 0.02)
Nurse intensity (per 1000 PD)	0.82 (−0.19 to 1.84)	0.08 (−1.35 to 1.51)
Patient volume	1.62 (−0.87 to 4.11)	−2.36 (−5.81 to 1.08)
Service availability		
Probability of operating cathlab/stroke unit	0.07 (0.01–0.13)	0.05 (−0.01 to 0.11)

Data represent difference-in-differences (DiD) estimates of the association between acquisition and patient health outcomes (excess and raw 30-day mortality; 30-day readmissions), staffing intensity, patient volume, and service availability (cardiac catheterization laboratory (cathlab) and stroke unit). Medical doctor (MD) and nurse intensity are calculated as the number of clinicians per 1000 patient-days (PD) (details in Appendix B). CI stands for confidence interval.

**Fig. 4:**
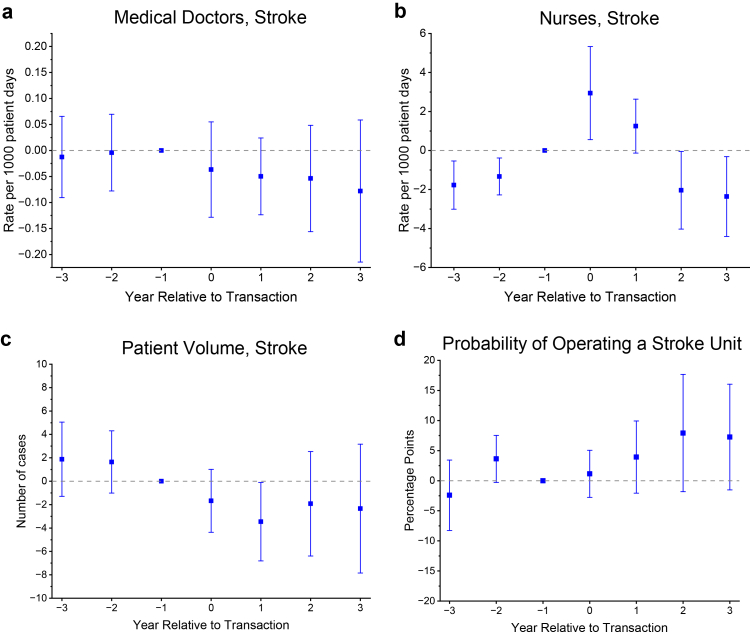
Secondary outcomes: Dynamic event-study estimates of the association between hospital acquisition and secondary outcomes for stroke. The panels illustrate difference-in-differences estimates for (a) medical doctor intensity, (b) nurse intensity, (c) stroke patient volume, and (d) the probability of operating a stroke unit. Estimates compare acquired hospitals to independent control hospitals, with the year prior to acquisition (Year −1) serving as the reference period. Error bars denote 95% confidence intervals.

For the primary outcome of stroke admissions, we found no significant average association between acquisition and changes in the excess 30-day mortality rate (0.43 pp decline, 95%CI from −1.38 to 0.53, p = 0.44) (Table 2). However, cohort-specific analyses revealed heterogeneity: acquisitions taking place in 2015 were associated with a 2.72pp decline (95%CI from −5.25 to −0.20, p = 0.03) by the third-year post-acquisition. Analysis of secondary outcomes for stroke showed that acquisition was associated with a 5.06 pp increase in the probability of providing stroke units, though this estimate did not reach statistical significance (95% CI from −0.82 to 10.94, p = 0.09) (Fig. 4). Nurse staffing intensity for stroke exhibited a volatile pattern. A significant pre-acquisition deficit reversed into a sharp increase in the year of acquisition (2.94 per 1000 patient days, 95% CI from 0.56 to 5.33, p = 0.02). However, this increase was transient, with staffing levels declining significantly by the second year (−2.04, 95% CI from −4.03 to −0.05, p = 0.05) and third year post-acquisition (−2.36, 95% CI from −4.40 to −0.31, p = 0.02).

Regarding secondary clinical outcomes, raw 30-day mortality rates declined in the second- and third-year post-acquisition for AMI admissions (Supplementary Figure S2). Conversely, we found no significant effect of hospital acquisitions on raw 30-day mortality rates for stroke, nor on 30-day readmission rates for both AMI and stroke (Supplementary Figures S2 and S3). Furthermore, evaluating the competing hypothesis of cost-cutting, we found no significant association between hospital acquisition and the probability of service line discontinuation for either AMI or stroke (Supplementary Figure S4).

Estimates obtained using entropy balancing to adjust for baseline covariates were highly consistent with the primary difference-in-differences results (Supplementary Figures S5–S9, Supplementary Table S2), confirming the robustness of our main findings. Cohort-specific analyses (Supplementary Figures S10 and S11, Supplementary Table S3) demonstrated a longitudinal shift in outcomes, with later acquisitions—specifically the 2015 cohort—demonstrating a significant association with improved quality of care for stroke compared with earlier cohorts. Heterogeneity analyses revealed similar post-acquisition associations across hospitals with both low and high bed utilization (Supplementary Figure S12, Supplementary Table S3). Stratification by baseline market competition revealed that the improvement in AMI mortality was significant even in markets with low competition (Supplementary Figure S13, Supplementary Table S3). In our sensitivity analysis subdividing the control group by system affiliation (Supplementary Figures S14–S17, Supplementary Table S4), distinct patterns emerged regarding resource allocation mechanisms. The increase in medical doctor intensity for AMI was more pronounced when evaluated against independent, non-system controls, whereas the increase in nurse intensity was more significant when compared to system-affiliated controls. Notably, when utilizing system-affiliated hospitals as the comparative baseline, the average treatment effect on the probability of operating a stroke unit became statistically significant.

Sensitivity analyses examining spillover effects on neighboring hospitals (competitors within 8 km and 15 km) revealed distinct patterns for AMI and stroke (Supplementary Table S5). For AMI, we found no statistically significant spillover effects on competitors' mortality, patient volume, or staffing intensity. Conversely, for stroke, competitors within an 8 km radius exhibited a significant increase in medical doctor intensity (0.14 per 1000 patient days, 95% CI from 0.01 to 0.27, p = 0.03) alongside a significant decline in the probability of operating a stroke unit (−3.99 pp, 95% CI from −6.07 to −1.90, p < 0.001). Despite these shifts in resource allocation, there were no significant spillover effects on competitors' excess stroke mortality (p = 0.21). When expanding the analysis to a 15 km radius, the results remained qualitatively similar; however, the magnitudes of these associations were generally attenuated, as expected with increased geographic distance.

## Discussion

In this study examining the impact of hospital acquisitions in a universal healthcare system with fixed prices, we found that acquisitions were associated with significantly reduced excess 30-day mortality rates for AMI admissions. This clinical improvement coincided with tangible investments in infrastructure, specifically a significant expansion of cardiac catheterization laboratories, accompanied by increased medical staffing intensity. While the average association for stroke mortality was not statistically significant, cohort-specific analyses revealed a temporal shift, with the 2015 cohort demonstrating significant quality improvements. This cohort-specific improvements aligns with a watershed moment in stroke care; in 2015, landmark clinical trials established endovascular mechanical thrombectomy as the new, highly effective standard of care for large-vessel ischemic stroke.^26^^,^^27^ Because implementing this complex intervention requires capital investment in neuro-angiography suites and highly specialized interventionalists, acquiring hospital systems may have been uniquely positioned—and sufficiently capitalized—to rapidly adopt these new guidelines compared to independent counterparts. Taken together, these findings suggest that in a fixed-price environment, hospital consolidation can facilitate quality gains through capital-intensive investment—particularly for high-volume, standardized conditions—though the realization of these benefits appears to depend on the timing of integration.

The divergence between AMI and stroke outcomes is noteworthy and likely reflects differences in clinical production functions. AMI care is highly standardized and dependent on specific, localized infrastructure (e.g., catheterization labs), making it amenable to rapid quality improvements through capital injection post-acquisition, as these outcomes may respond more directly to infrastructure investment.^28^ In contrast, stroke care requires broader multidisciplinary coordination and complex, time-sensitive pathways that may be more difficult to optimize during the organizational upheaval of an acquisition,^29^ highlighting that stroke care relies more fundamentally on coordination and process quality. This distinction implies that while acquisitions provide a foundation for improvement through financial stability, achieving quality gains in complex, labor-intensive service lines like stroke may require more targeted, condition-specific integration strategies than simple capital investment.

Our results support the theoretical “negative margin” framework,^9^^,^^20^ where consolidation in fixed-price systems enables quality improvement through investment rather than market power. Importantly, our data suggest that these gains were driven by resource expansion rather than cost-cutting, as we found no evidence of acquired hospitals discontinuing their baseline AMI or stroke service lines. Crucially, we found that AMI mortality improved even in markets with high baseline concentration (low competition). This finding contrasts with standard market power theories—which typically predict quality declines in the absence of competitive pressure—and reinforces the hypothesis that in this specific regulatory setting, quality gains are driven by the acquired hospitals' enhanced capacity for capital investment. The observed expansion of cardiac catheterization laboratories serves as a plausible mechanism for the reduction in AMI mortality.

The differential changes in staffing observed in the year immediately preceding acquisition likely reflect anticipatory organizational restructuring or shifts in patient volume following the announcement of an acquisition. Our results, which were robust to the use of entropy balancing to address potential selection bias, remain qualitatively similar, confirming that the post-acquisition improvements were not driven by baseline hospital characteristics.

A key strength of our study is the use of a rigorous dynamic difference-in-differences event study framework with staggered adoption, accounting for heterogeneous treatment effects over time. We focused on high-volume emergency services (AMI and stroke) to minimize the risk of patient selection bias and to ensure hard, reliable measures of quality.

Our findings offer practical insights for regional care organization and clinical leadership. From a planning perspective, hospital consolidation may be specifically encouraged in regions where standalone hospitals lack the capital reserves necessary to provide high-level interventional infrastructure for acute cardiovascular conditions. In such settings, acquisition may act as a vehicle for technological catch-up. For clinicians and hospital managers, the practical implication is that while ownership changes provide a strategic window to secure new capital and staffing, they also require ‘coordination-aware’ management. Clinical leadership must proactively safeguard multidisciplinary pathways during the post-acquisition transition to ensure that organizational restructuring does not disrupt the complex, time-sensitive integration required for effective stroke care.

Our study also has several limitations. First, our results represent the average effect of hospital acquisitions, which may obscure heterogeneous impacts across individual transactions. Specifically, while our manual web search identified the occurrence of ownership changes and we employed entropy balancing to minimize baseline structural differences across important observables, we were unable to systematically capture the underlying reasons for these acquisitions. These motivations could vary greatly, ranging from a shortage of skilled workers or management errors to a changing regional supply landscape. Differentiating these underlying factors represents an important avenue for future research to further elucidate the mechanisms driving the relationship between hospital acquisitions and quality of care. Second, while we performed extensive sensitivity analyses, including the exclusion of local competitor hospitals within 15 km of acquired hospitals, the potential for unobserved positive spillover effects on remaining control hospitals remains. Such spillovers would lead to an attenuation of our difference-in-differences estimates toward the null, suggesting our reported effect size for AMI may be a conservative estimate. Third, by focusing on mortality for emergency services, we did not examine other critical dimensions of quality, such as process quality (e.g., time to treatment) or patient experience. Finally, although we employed entropy balancing to mitigate the potential for selection bias inherent in voluntary treatment (acquisition), we cannot completely rule out all forms of time-varying endogeneity. While new clinical guidelines were introduced universally during our study period, there may be unobserved differences in the speed at which individual facilities adopted these shifts—such as the introduction of mechanical thrombectomy for stroke^26^^,^^27^ and the transition toward radial access for percutaneous coronary interventions, which significantly reduced mortality and bleeding complications.^30^

This study demonstrates that hospital acquisitions within a fixed-price, universal healthcare system were associated with significant improvements in clinical outcomes for AMI, likely mediated by increased medical staffing intensity and the expanded availability of advanced diagnostic technology. Importantly, these gains were achieved without adverse effects on the quality of stroke care, which showed a positive trajectory in more recent cohorts. As noted, this temporal improvement likely reflects the enhanced capacity of acquiring networks to rapidly invest in emerging, capital-intensive treatments like mechanical thrombectomy. These findings suggest that hospital consolidation can drive clinical excellence through infrastructure investment, even in regulatory environments that preclude market power-driven price increases. However, the variation in results between AMI and stroke suggests that while acquisitions may provide a foundation for improvement, achieving universal quality gains across diverse clinical conditions likely requires targeted, condition-specific integration strategies.

## Contributors

EEB: conceptualization, data curation, formal analysis, funding acquisition, investigation, methodology, project administration, visualization, writing—original draft. RB: conceptualization, funding acquisition, methodology, project administration, writing—review & editing. JS: conceptualization, funding acquisition, investigation, methodology, project administration, writing—review & editing.

EEB, RB, and JS verified the data and had access to raw data. JS had final responsibility for decision to submit for publication.

## Data sharing statement

Data on hospital characteristics were derived from publicly available German structural quality reports and hospital directories. However, the data on hospital quality indicators (including mortality and readmission rates) were provided by the Scientific Institute of the AOK (WIdO). Due to data protection regulations and data use agreements, the authors are not permitted to share the WIdO data publicly. Researchers wishing to access the quality indicator data must apply directly to WIdO. Code used for the statistical analysis, along with a comprehensive data dictionary, will be made available.

## Declaration of AI and AI-assisted technologies in the writing process

During the preparation of this work, the authors used Gemini for English grammar checks. After using this tool/service, the authors reviewed and edited the content as needed and take full responsibility for the content of the publication.

## Declaration of interests

All authors (EEB, RB, and JS) report support for the present manuscript from the German Research Foundation (funding for staff and travel). Outside the submitted work, RB reports grants or contracts paid to his institution from the World Health Organization, the German Innovation Fund, and the ECDC; consulting fees from Techniker Krankenkasse, GKV Spitzenverband, and Medizinischer Dienst; payment or honoraria from Stadt Ellwangen, Evangelische Bank, and Agenon; and an unpaid leadership role as a Member of the Government Commission on Hospital Reform (2022–2025). JS reports a paid leadership role as the Deputy Chair of the German Council of Experts in Health and Care at the German Federal Ministry of Health. EEB declares no other competing interests.

## Appendix A Supplementary data

Supplementary Material

Translated Abstract
