# Supplementary material for: Hospital acquisitions and 30-day mortality after acute myocardial infarction and stroke in Germany: a quasi-experimental cohort study
Source: Lancet Reg Health Eur. 2026 Jul 23;68:101788. doi: 10.1016/j.lanepe.2026.101788 (PMC13425888; doi:10.1016/j.lanepe.2026.101788)
Supplement: Supplementary Material [file mmc1.pdf]

Supplementary Material

Hospital acquisitions and 30-day mortality after acute myocardial infarction and stroke in Germany: a quasi-experimental cohort study

Esra Eren Bayindir, PhD[1]; Reinhard Busse, MD[2]; Jonas Schreyögg, PhD[1]

[1]Hamburg Center for Health Economics, University of Hamburg, Hamburg, Germany
[2] Department of Health Care Management, Berlin University of Technology, Berlin, Germany

Table of Contents

Appendix A 3

Information on quality indicators 3

Appendix B 4

Details on secondary outcomes 4

Appendix C 5

Entropy Balancing 5

Supplementary Figures 6

Supplementary Figure 1 6

Supplementary Figure 2 7

Supplementary Figure 3 7

Supplementary Figure 4- 7

Supplementary Figure 5 8

Supplementary Figure 6 8

Supplementary Figure 7 9

Supplementary Figure 8 9

Supplementary Figure 9 10

Supplementary Figure 10 11

Supplementary Figure 11 12

Supplementary Figure 12 13

Supplementary Figure 13 14

Supplementary Figure 14 15

Supplementary Figure 15 16

Supplementary Figure 16 17

Supplementary Figure 17 18

Supplementary Tables 19

Supplementary Table 1 19

Supplementary Table 2 20

Supplementary Table 3 21

Supplementary Table 4 22

Supplementary Table 5 23

Appendix A

Information on quality indicators
Risk adjustment was performed using logistic regression.

Inclusion criteria and risk adjustment factors of health outcome indicators

Acute Myocardial Infarction (AMI)

Definition: Patients older than 30 years of age with heart attack main diagnoses.

Inclusion diagnosis ICD-10-German Modification codes (main diagnoses): I21, I22.

Risk factors: Age, sex, type of myocardial infarction (acute transmural myocardial infarction of the anterior wall, acute transmural myocardial infarction of the posterior wall, acute transmural myocardial infarction at other locations, acute subendocardial myocardial infarction, acute transmural myocardial infarction at unspecified location, other myocardial infarction), atherosclerosis, dilated cardiomyopathy, heart failure, cardiogenic and NOS shock, 3rd degree atrioventricular block, ventricular tachycardia, ventricular fibrillation/flutter, cerebral infarction, intracerebral hemorrhage, status post stroke or cerebral hemorrhage, severe kidney disease, malignant neoplasm, metastases.

Stroke

Definition: Patients older than 30 years of age with cerebral infarction or intracerebral hemorrhage main diagnoses.

Inclusion diagnosis ICD-10-German Modification codes (main diagnoses): I61, I63, I64.

Risk factors: Age, sex, type of stroke (intracerebral hemorrhage, cerebral infarction, stroke-not referred to as hemorrhage or infarction), atrial fibrillation/flutter, atherosclerosis, congestive heart failure, dilated cardiomyopathy, 3rd degree AV block, aortic and mitral valve defects, ventricular tachycardia, atherosclerosis of the limb arteries, severe kidney disease, chronic renal failure, malignant neoplasms, metastases.

Appendix B

Details on secondary outcomes

To explore potential mechanisms driving the findings on excess 30-day mortality rates for acute myocardial infarction (AMI) and stroke, we investigated changes in the number of medical doctors and nurses per 1000 patient day; changes in patient volume for AMI and stroke patients; and changes in the presence of cardiac catheterization laboratories and stroke units. Number of medical doctors and nurses per 1000 patient day were constructed using medical staff data by department and admissions by 4-digit ICD code by department from structured hospital quality reports, and average length of stay by 4-digit ICD codes from the federal statistical office.

To calculate the average care intensity (number of medical doctors and nurses per 1000 patient day) by department, we have used the average length of stays by four digit ICDs obtained from Federal Statistical Office of Germany (https://www.statistischebibliothek.de/mir/receive/DESerie_mods_00000950).

We first calculated the average care intensity (staff per 1000 patient days) for each department in the hospitals since acute myocardial infarction or stroke patients can be admitted to different departments. This calculation combined the departmental staff figures with the patient days, derived from the departmental admissions and the average length of stay per 4-digit ICD code.

After calculating the care intensity by department, we calculated the medical doctor and nurse intensity for AMI and stroke patients by taking the weighted average of care intensity by department, where the number of AMI and stroke cases were used as weights, respectively.

Appendix C

Entropy Balancing

Even though we find no evidence for pre-trends in general, to address the concern that acquired hospitals can be different from the hospitals that did not experience any change in ownership, we performed entropy balancing. We used the number of hospital beds, the number of acute myocardial infarction (AMI) and stroke cases, an exogenous measure of hospital competition for AMI and stroke and bed utilization rate for entropy balancing. As an exogenous measure of hospital competition, we used predicted Herfindahl Hirschman Index (HHI), which we calculated following Kessler and Mcclellan (2000),[1] Gowrisankaran and Town (2003).[2] We utilized the bed utilization rate as a proxy for potential hospital profitability. This was preferred over traditional financial indicators (e.g., EBITDA per bed) due to high rates of missing data in financial metrics, the usage of which would have introduced sample selection bias, particularly concerning public hospitals in Germany.

References

1. Kessler DP, McClellan MB. Is hospital competition socially wasteful? *Q J Econ* 2000; **115**: 577–615.
2. Gowrisankaran G, Town RJ. Competition, payers, and hospital quality. *Health Serv Res* 2003; **38**: 1403–22.

Supplementary Figures

Supplementary Figure 1- Unadjusted temporal trends of clinical outcomes for acute myocardial infarction (AMI) and stroke (2009–2019). The graphs display the unadjusted annual mean values for (a) excess 30-day mortality, (b) raw 30-day mortality, and (c) 30-day readmission rates for AMI (left column) and stroke (right column) admissions throughout the study period. Grey square markers denote the control group, and red circle markers denote the treatment group (acquired hospitals). Error bars represent 95% confidence intervals.

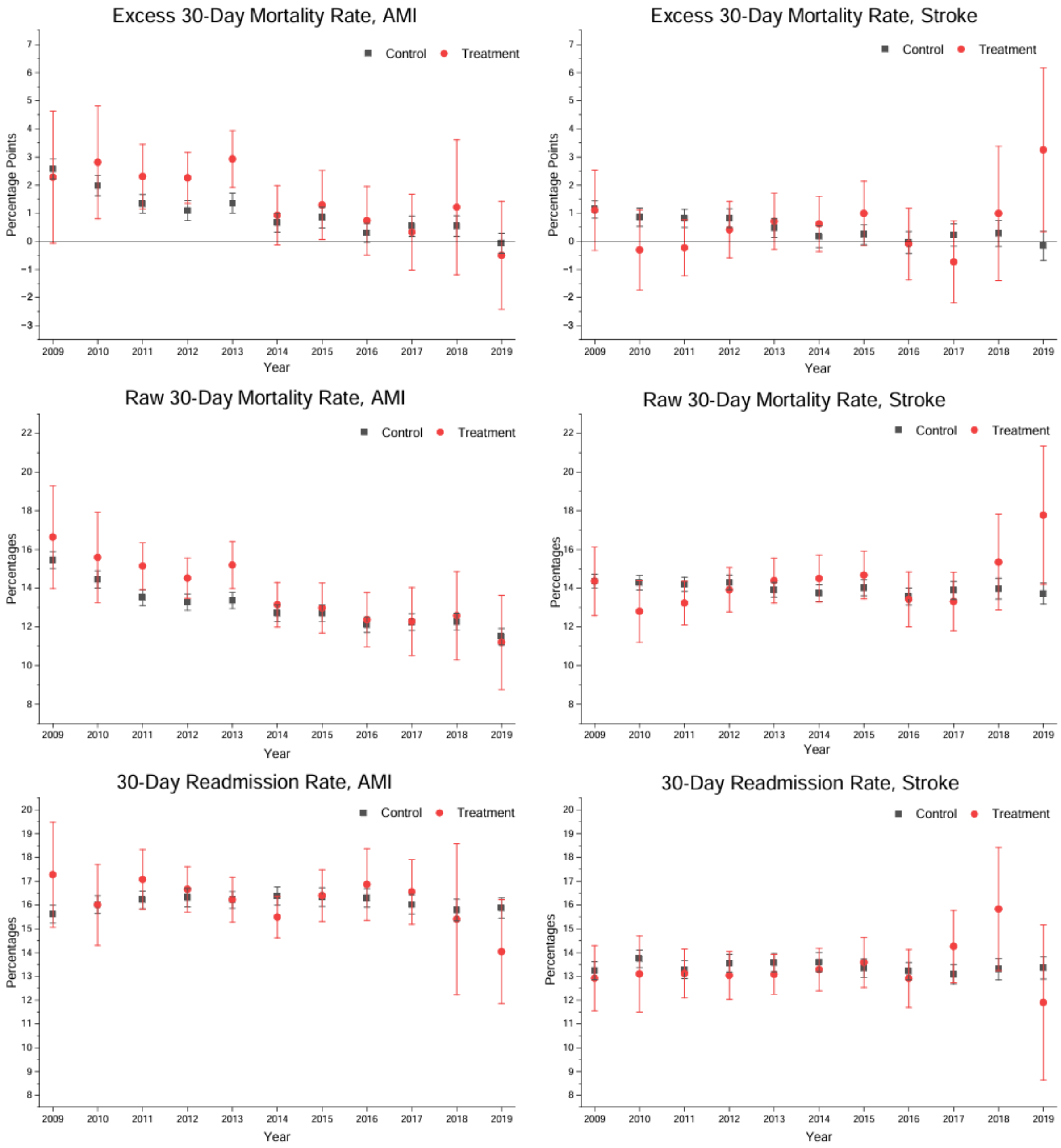

Supplementary Figure 2- Dynamic difference-in-differences event study estimates of acquisition effects on raw 30-day mortality and readmission rates for acute myocardial infarction (AMI). Error bars denote 95% confidence intervals.

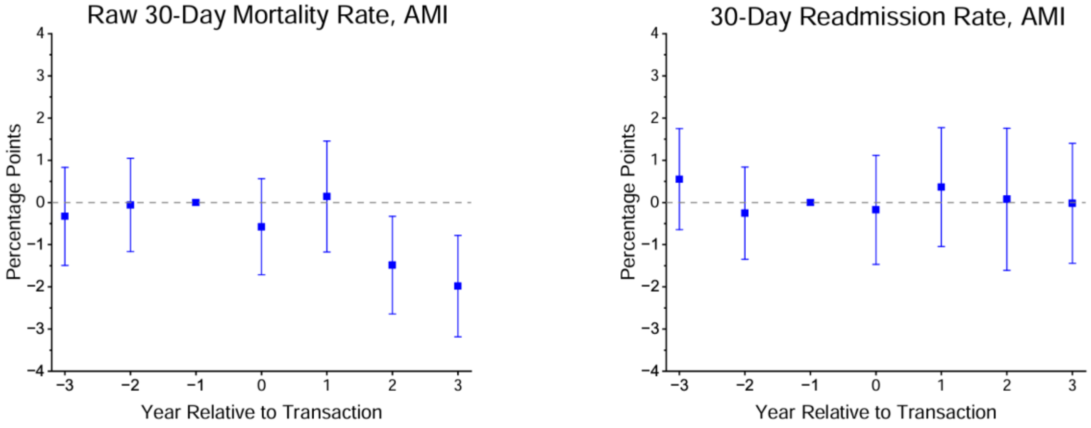

Supplementary Figure 3- Dynamic difference-in-differences event study estimates of acquisition effects on raw 30-day mortality and readmission rates for stroke. Error bars denote 95% confidence intervals.

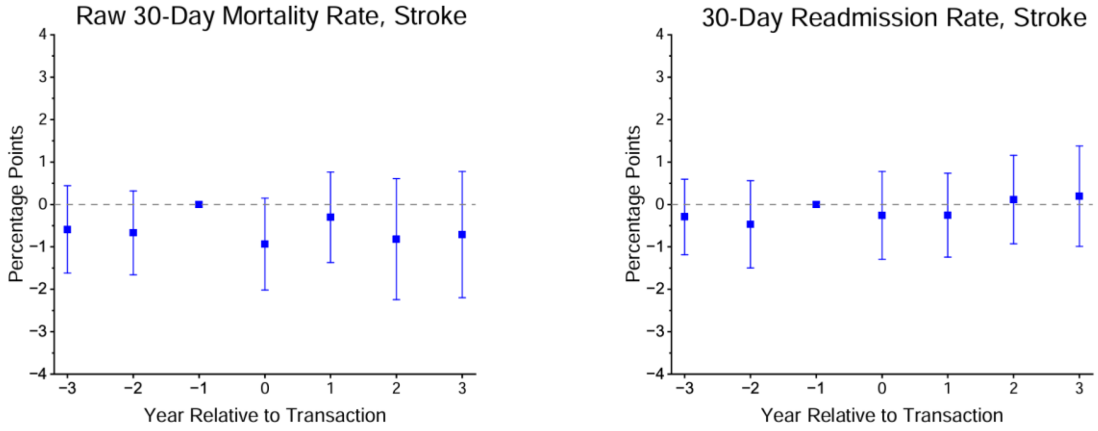

Supplementary Figure 4- Probability of service line discontinuation: Dynamic event-study estimates of the association between hospital acquisition and the cessation of acute myocardial infarction (AMI) and stroke care. The panels display difference-in-differences estimates evaluating the hypothesis of post-acquisition cost-cutting through service reduction. Estimates represent the probability of an acquired hospital ceasing to treat AMI (left) or stroke (right) patients compared to control hospitals, with the year prior to acquisition (Year -1) serving as the reference period. Error bars denote 95% confidence intervals.

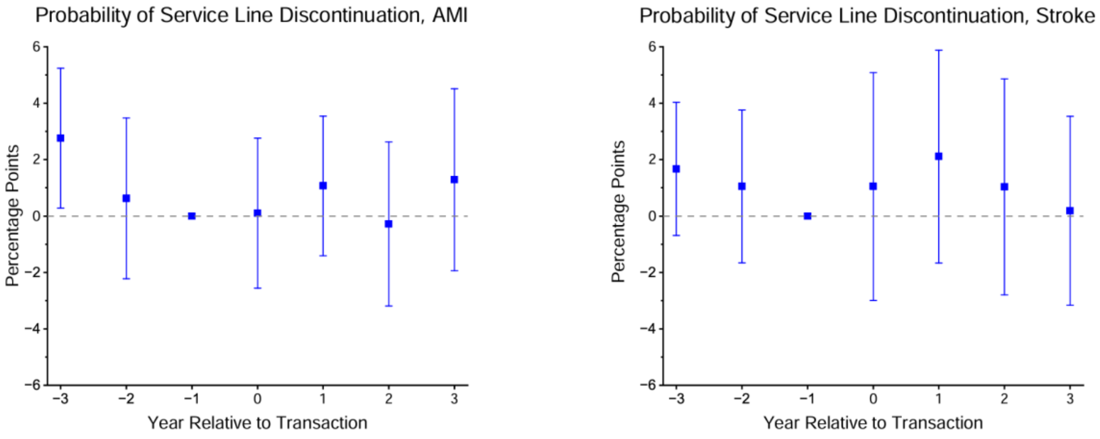

Supplementary Figure 5- Dynamic difference-in-differences event study estimates of acquisition effects on excess 30-day mortality rates for acute myocardial infarction (AMI) and stroke, with entropy balancing. Error bars denote 95% confidence intervals.

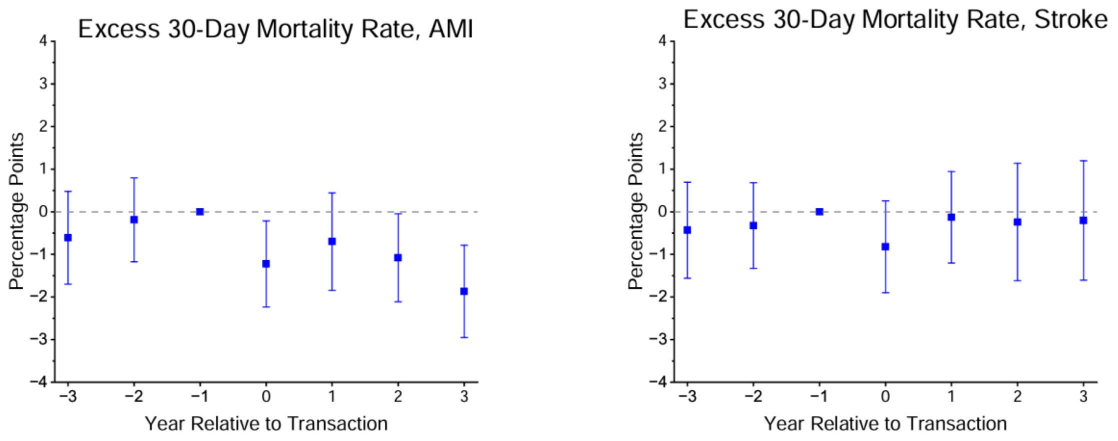

Supplementary Figure 6- Dynamic difference-in-differences event study estimates of acquisition effects on medical doctor and nurse intensity for acute myocardial infarction (AMI) care, AMI patient volume, and probability of operating a cardiac catheterization laboratory, with entropy balancing. Error bars denote 95% confidence intervals.

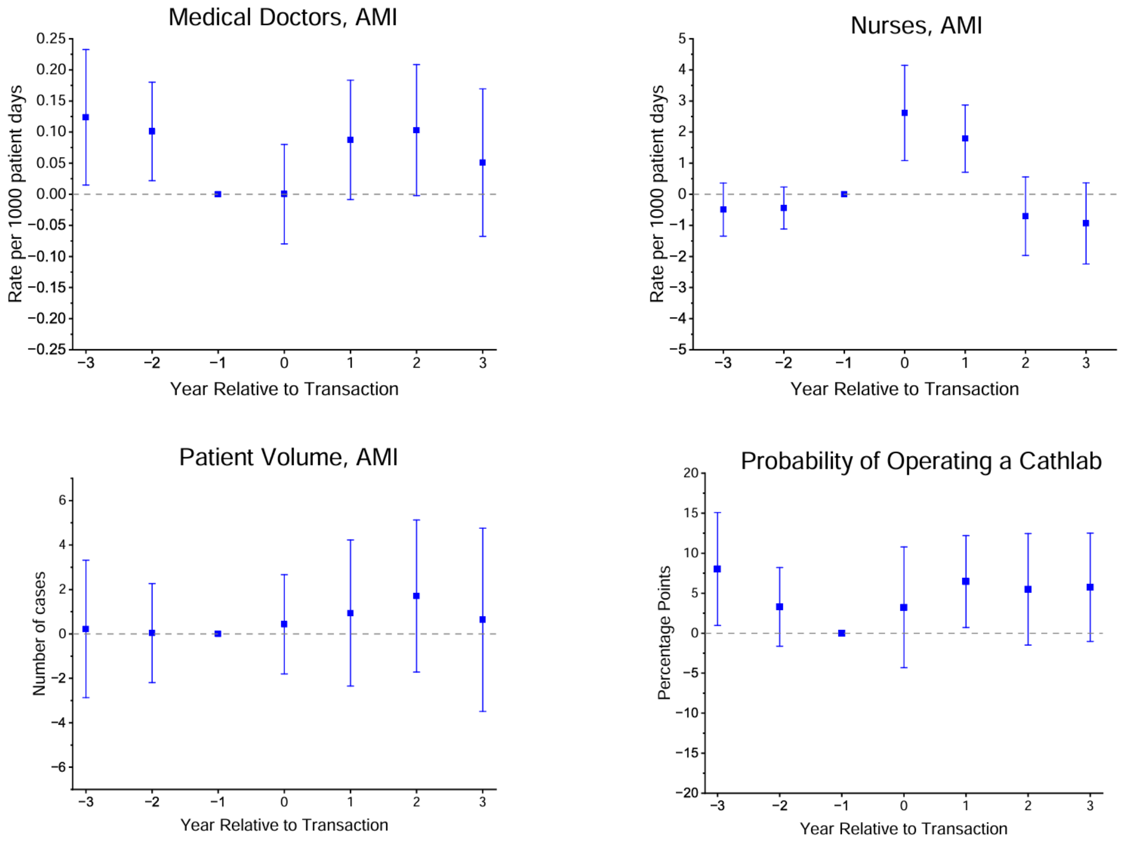

Supplementary Figure 7- Dynamic difference-in-differences event study estimates of acquisition effects on raw 30-day mortality and readmission rates for acute myocardial infarction (AMI), with entropy balancing. Error bars denote 95% confidence intervals.

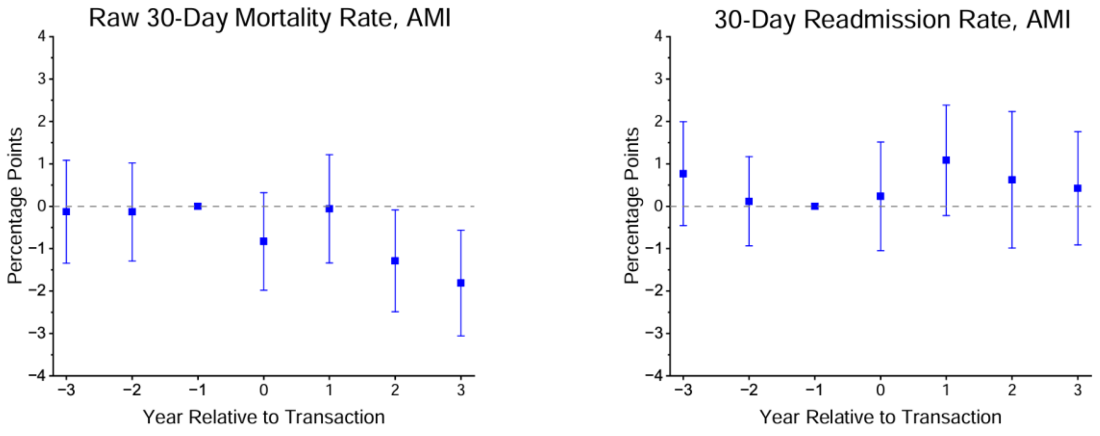

Supplementary Figure 8- Dynamic difference-in-differences event study estimates of acquisition effects on medical doctor and nurse intensity for stroke care, stroke patient volume, and probability of operating a stroke unit, with entropy balancing. Error bars denote 95% confidence intervals.

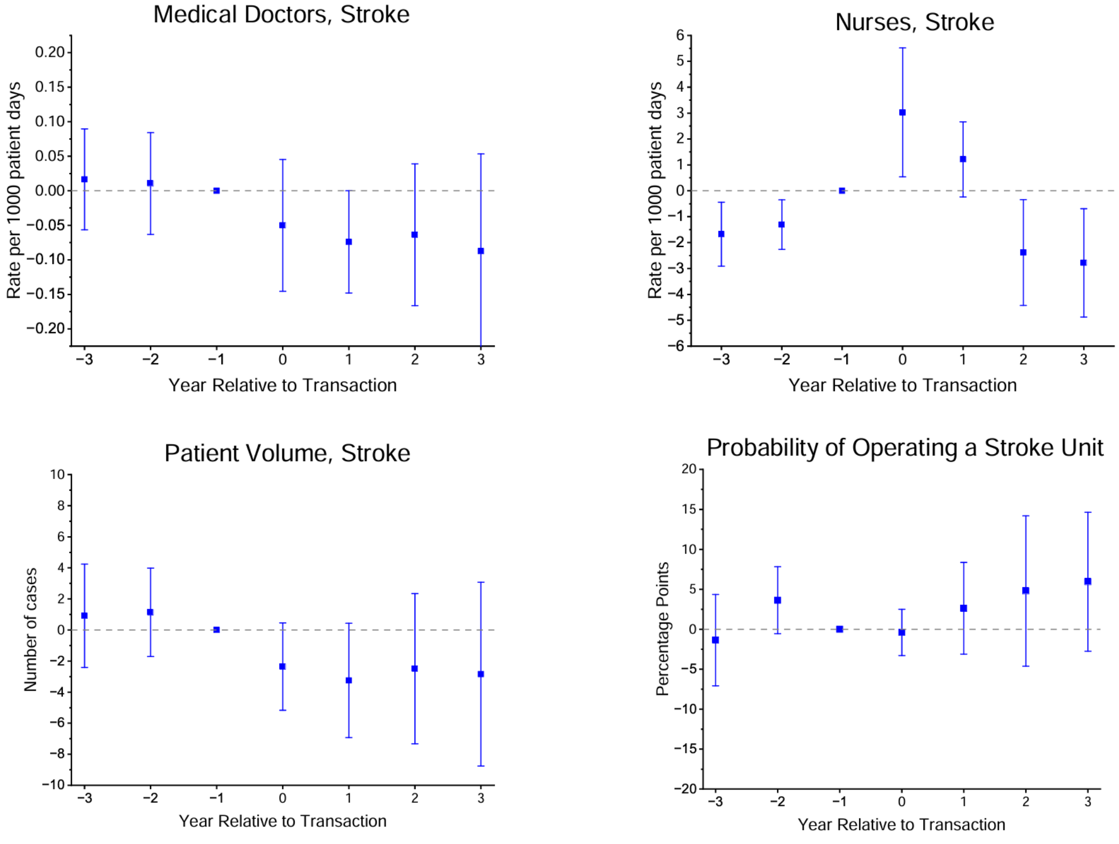

Supplementary Figure 9- Dynamic difference-in-differences event study estimates of acquisition effects on raw 30-day mortality and readmission rates for stroke, with entropy balancing. Error bars denote 95% confidence intervals.

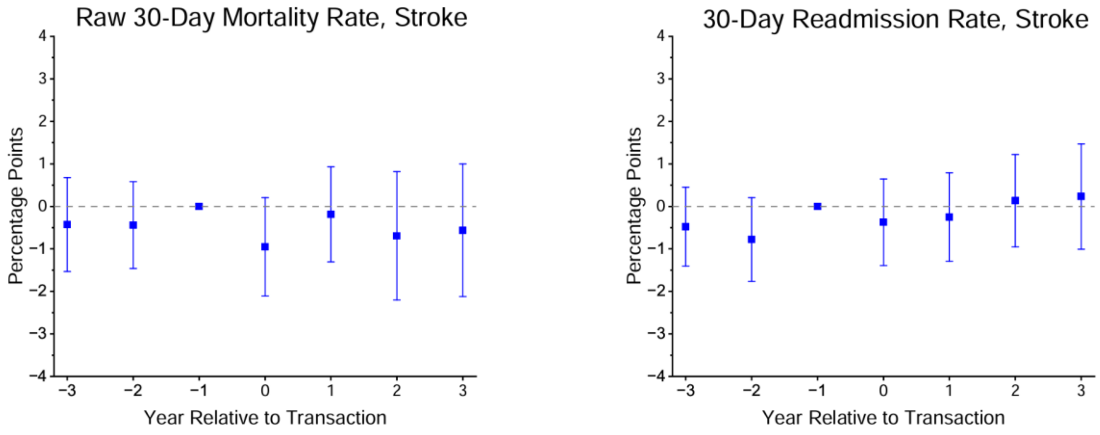

Supplementary Figure 10- Dynamic difference-in-differences event study estimates of acquisition effects on excess 30-day mortality for acute myocardial infarction (AMI), by acquisition cohort. Error bars denote 95% confidence intervals.

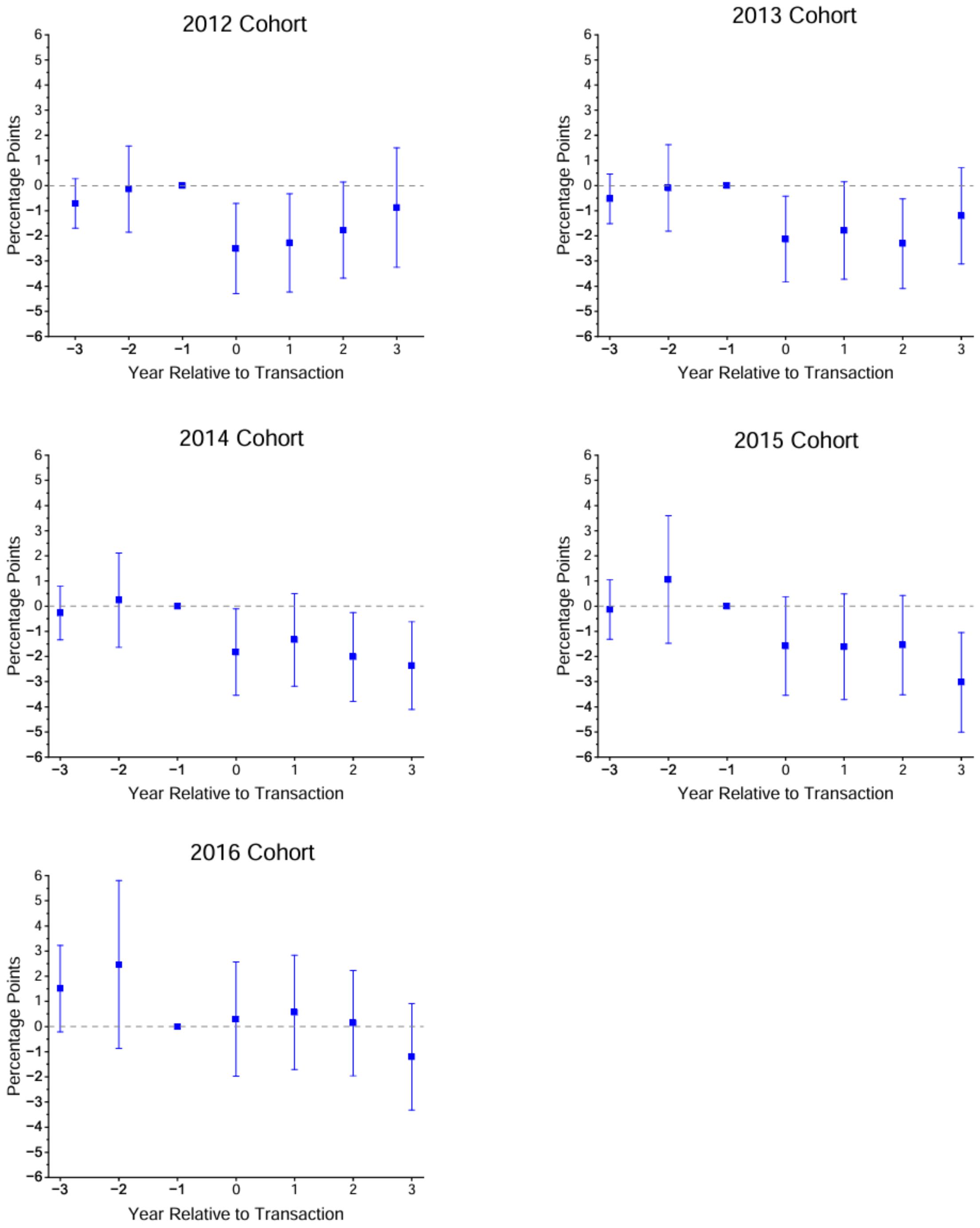

Supplementary Figure 11- Dynamic difference-in-differences event study estimates of acquisition effects on excess 30-day mortality for stroke, by acquisition cohort. Error bars denote 95% confidence intervals.

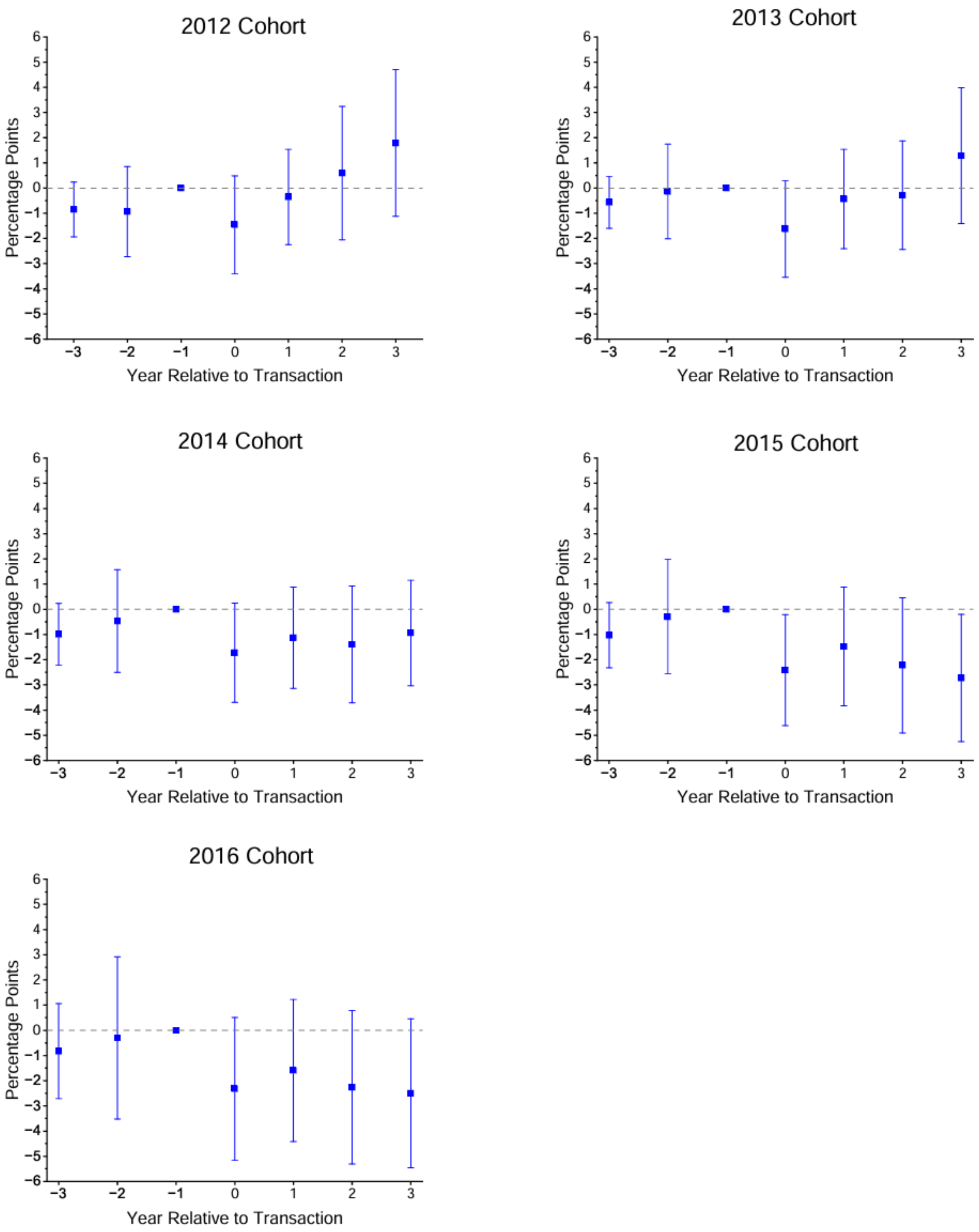

Supplementary Figure 12- Dynamic difference-in-differences event study estimates of acquisition effects on excess 30-day mortality for acute myocardial infarction (AMI) and stroke, by bed utilization rate. Error bars denote 95% confidence intervals.

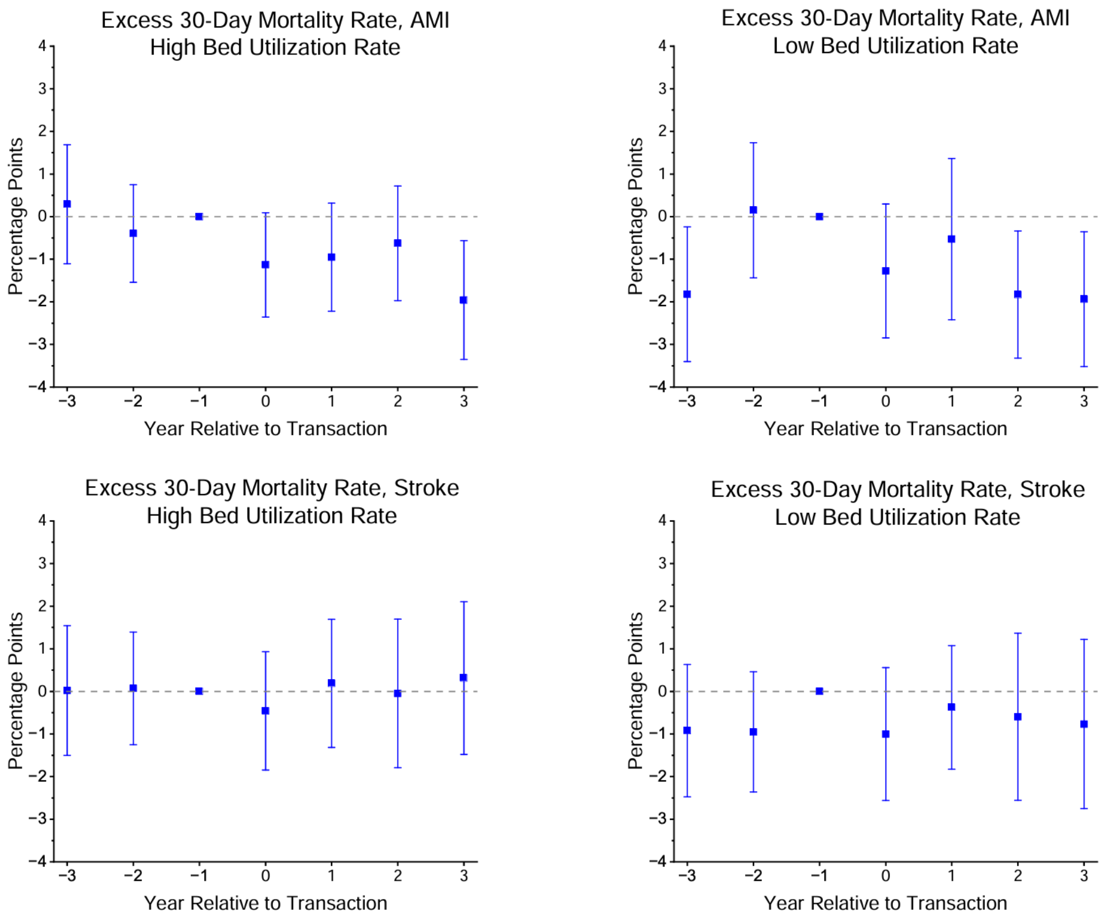

Supplementary Figure 13- Dynamic difference-in-differences event study estimates of acquisition effects on excess 30-day mortality for acute myocardial infarction (AMI) and stroke, by the intensity of hospital competition. Market competition is defined using the predicted Herfindahl-Hirschman Index (HHI) at baseline. Markets are classified as having high competition if the predicted HHI is ≤0.25, and low competition if the predicted HHI is >0.25. Error bars denote 95% confidence intervals.

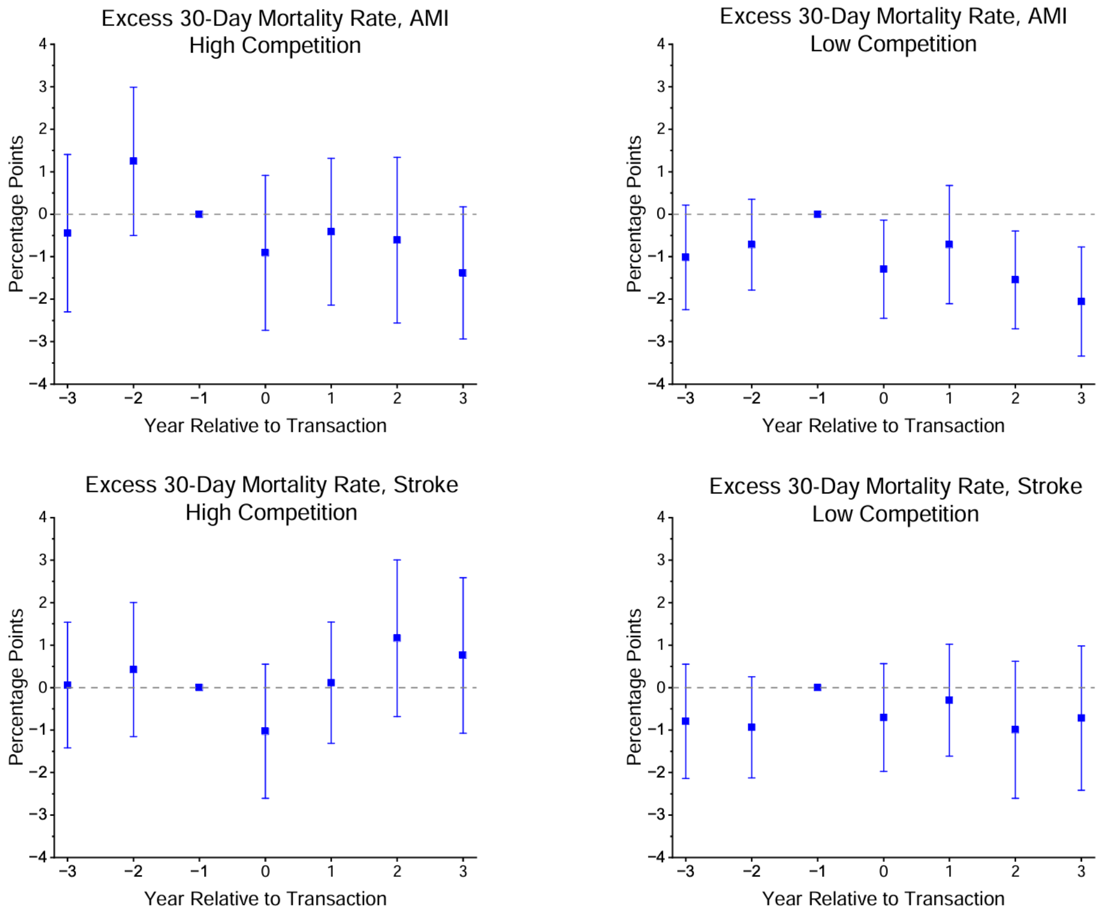

Supplementary Figure 14- Sensitivity analysis by control group system affiliation: Dynamic event-study estimates of the association between hospital acquisition and clinical outcomes for acute myocardial infarction (AMI). The panels display difference-in-differences estimates for excess 30-day mortality, raw 30-day mortality, and 30-day readmission rates for AMI. To account for underlying structural variations, estimates compare acquired hospitals to independent, non-system controls (left column) and system-affiliated controls (right column). The year prior to acquisition (Year -1) serves as the reference period. Error bars denote 95% confidence intervals.

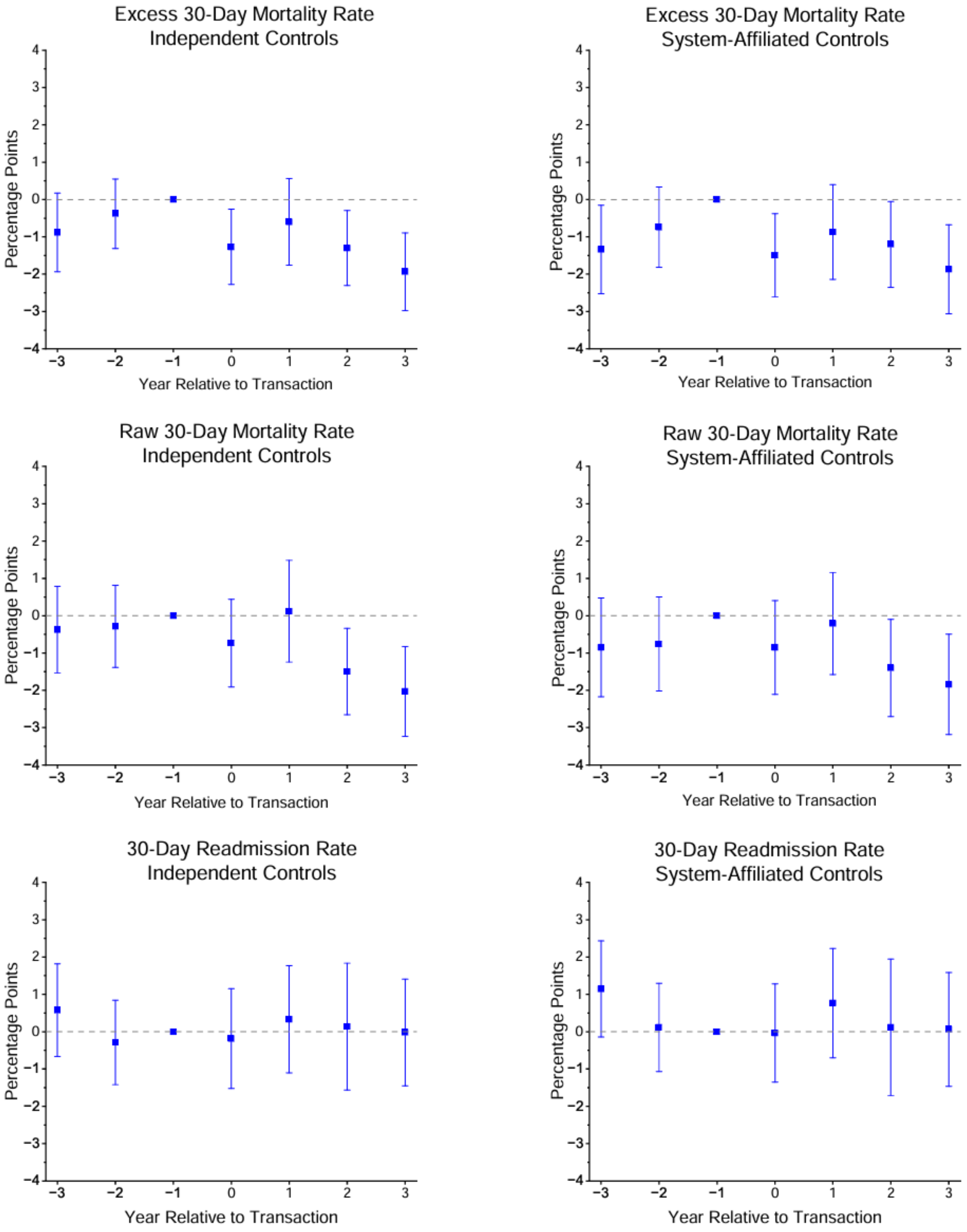

Supplementary Figure 15-Sensitivity analysis by control group system affiliation: Dynamic event-study estimates of the association between hospital acquisition and secondary outcomes for acute myocardial infarction (AMI). The panels display difference-in-differences estimates for medical doctor intensity, nurse intensity, AMI patient volume, and the probability of operating a cardiac catheterization laboratory. Estimates compare acquired hospitals to independent, non-system controls (left column) and system-affiliated controls (right column), with the year prior to acquisition (Year -1) serving as the reference period. Error bars denote 95% confidence intervals.

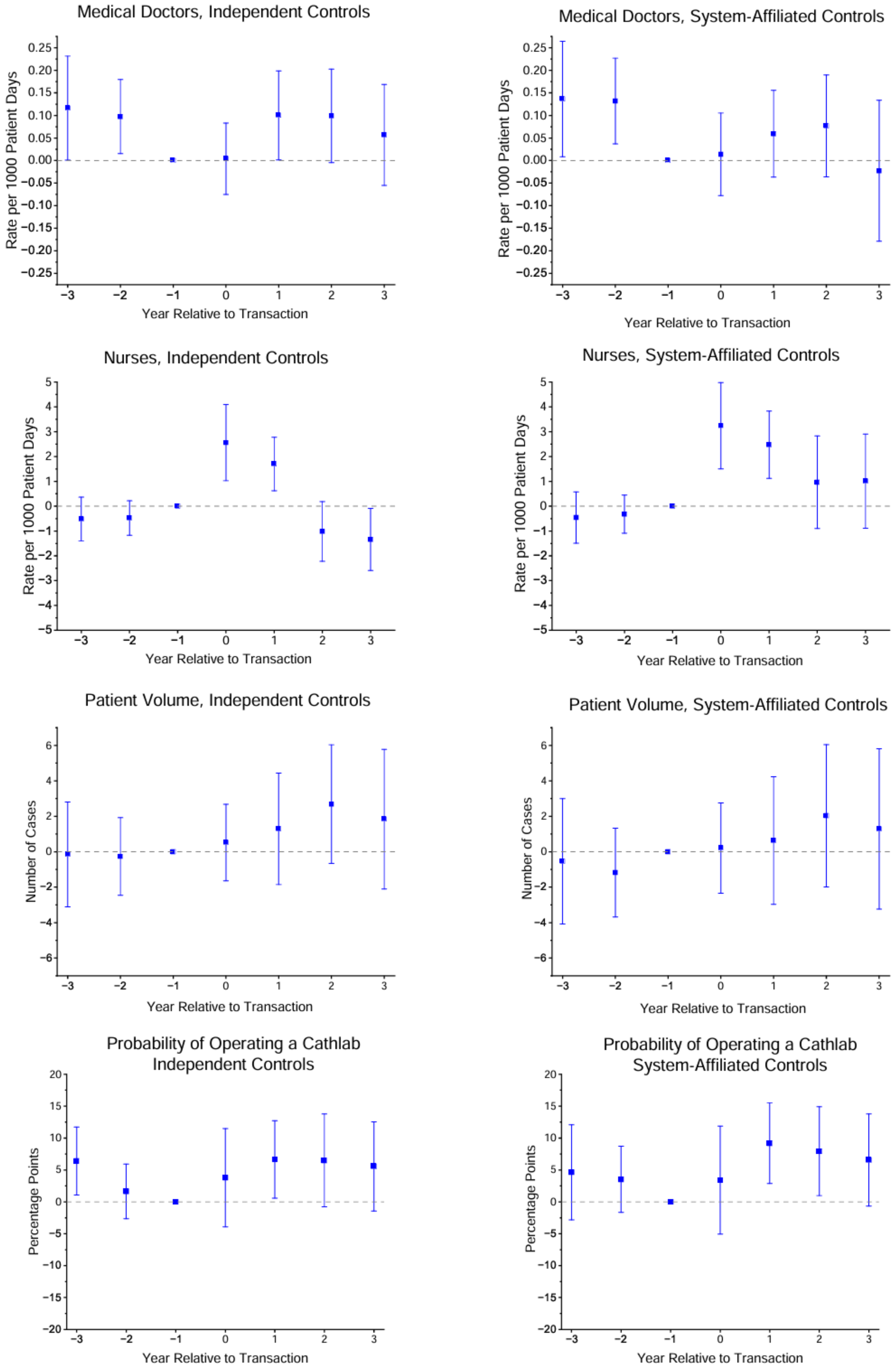

Supplementary Figure 16-Sensitivity analysis by control group system affiliation: Dynamic event-study estimates of the association between hospital acquisition and clinical outcomes for stroke. The panels display difference-in-differences estimates for excess 30-day mortality, raw 30-day mortality, and 30-day readmission rates for stroke. Estimates compare acquired hospitals to independent, non-system controls (left column) and system-affiliated controls (right column), with the year prior to acquisition (Year -1) serving as the reference period. Error bars denote 95% confidence intervals.

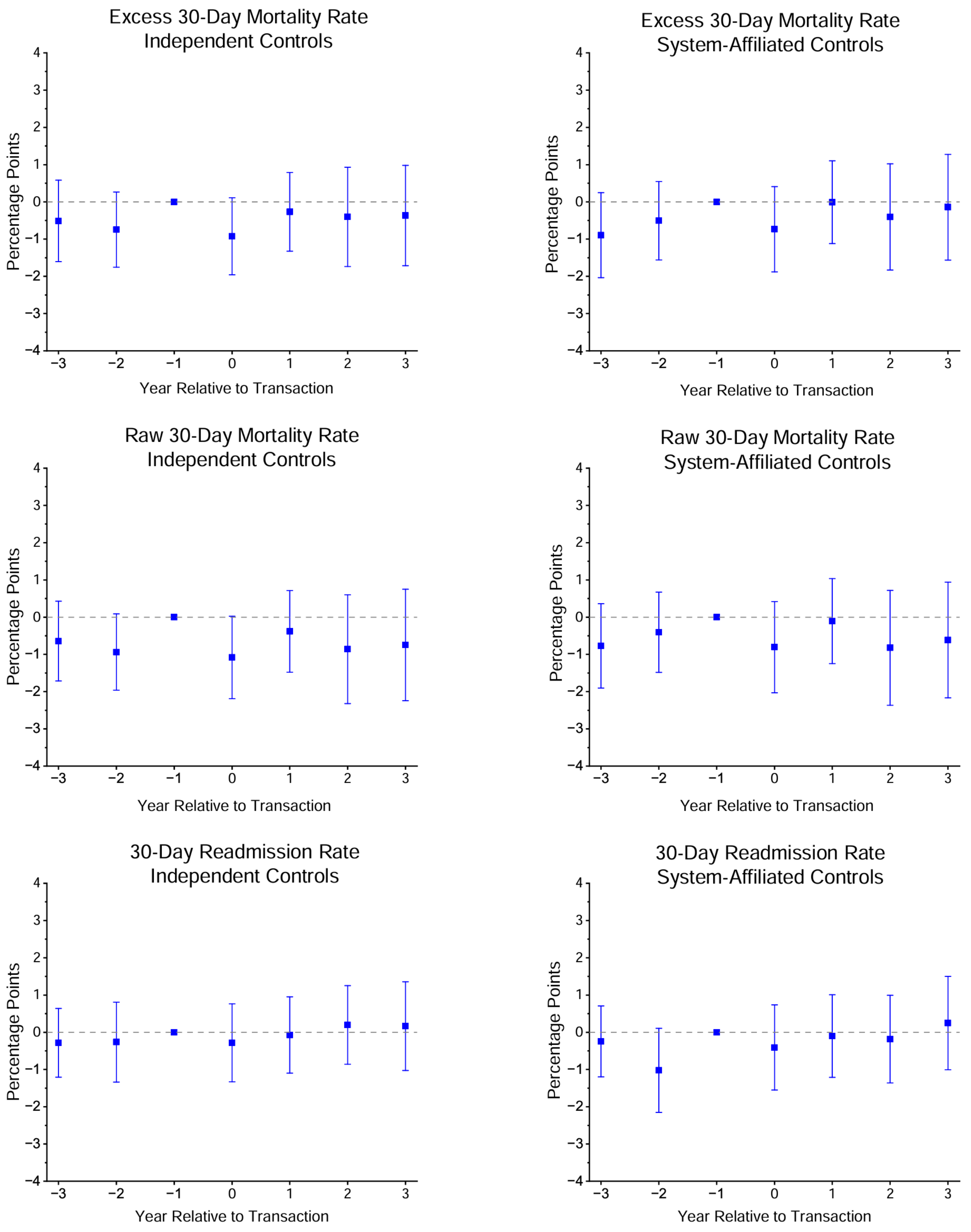

Supplementary Figure 17-Sensitivity analysis by control group system affiliation: Dynamic event-study estimates of the association between hospital acquisition and secondary outcomes for stroke. The panels display difference-in-differences estimates for medical doctor intensity, nurse intensity, stroke patient volume, and the probability of operating a stroke unit. Estimates compare acquired hospitals to independent, non-system controls (left column) and system-affiliated controls (right column), with the year prior to acquisition (Year -1) serving as the reference period. Error bars denote 95% confidence intervals.

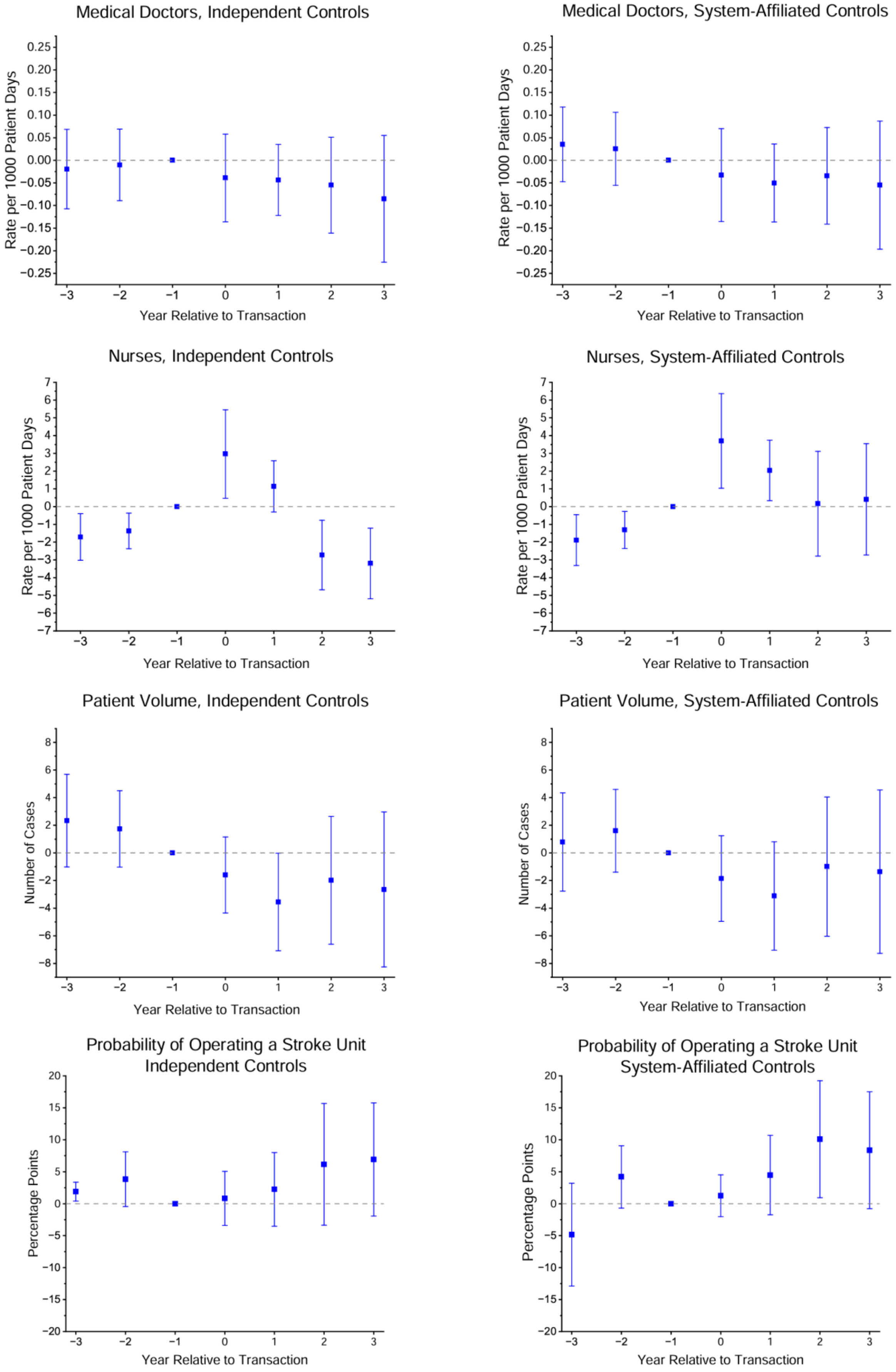

Supplementary Tables

Supplementary Table 1- Tests of the parallel trends assumption. The reported pre-trend P-values test the joint hypothesis that the pre-transaction coefficients ($t = -3$ and $t = -2$) from the dynamic event study are jointly zero. AMI indicates acute myocardial infarction; Cathlab, cardiac catheterization laboratory.

	P-value	
**Outcomes**	**AMI**	**Stroke**
Primary outcomes		
Excess 30-day mortality	0·24	0·44
Secondary outcomes		
Raw 30-day mortality	0·83	0·36
30-day readmission rate	0·39	0·66
Staffing and volume		
MD intensity (per 1000 PD)	0·03	0·95
Nurse intensity (per 1000 PD)	0·42	0·02
Patient volume	0·84	0·44
Service availability		
Probability of operating cathlab/stroke unit	0·06	0·14

Supplementary Table 2- Difference-in-differences estimates of hospital acquisition on clinical outcomes, staffing, and service availability for acute myocardial infarction (AMI) and stroke, with entropy balancing. Data represent difference-in-differences estimates of the association between acquisition and patient health outcomes (excess and raw 30-day mortality; 30-day readmissions), staffing intensity, patient volume, and service availability (cardiac catheterization laboratory and stroke unit). Medical doctor (MD) and nurse intensity are calculated as the number of clinicians per 1000 patient-days (PD) (details in Appendix B). CI stands for confidence interval. The reported pre-trend P-values test the joint hypothesis that pre-transaction coefficients from the dynamic event study are jointly zero.

	**Acute Myocardial Infarction**		**Stroke**	
**Outcome**	**DiD Estimate (95% CI)**	**Pre-trend P-value**	**DiD Estimate (95% CI)**	**Pre-trend P-value**
Primary outcomes				
Excess 30-day mortality	-0·012 (-0·020 to -0·004)	0·54	-0·004 (-0·014 to 0·007)	0·73
Secondary outcomes				
Raw 30-day mortality	-0·010 (-0·019 to 0·000)	0·97	-0·006 (-0·017 to 0·005)	0·36
30-day readmission rate	0·006 (-0·005 to 0·017)	0·44	-0·001 (-0·009 to 0·007)	0·3
Staffing and volume				
MD intensity (per 1000 PD)	0·06 (-0·02 to 0·14)	0·03	-0·07 (-0·15 to 0·01)	0·91
Nurse intensity (per 1000 PD)	0·78 (-0·25 to 1·80)	0·41	-0·09 (-1·57 to 1·39)	0·02
Patient volume	0·92 (-1·73 to 3·58)	0·99	-2·74 (-6·49 to 1·02)	0·74
Service availability				
Probability of operating cathlab/stroke unit	0·05 (-0·00 to 0·11)	0·08	0·03 (-0·02 to 0·08)	0·21

Supplementary Table 3- Heterogeneity of difference-in-differences estimates of hospital acquisition on excess 30-day mortality rate for acute myocardial infarction (AMI) and stroke by cohort, bed utilization rate, and market competition intensity. CI stands for confidence interval. The reported pre-trend P-values test the joint hypothesis that pre-transaction coefficients from the dynamic event study are jointly zero.

	**Acute Myocardial Infarction**		**Stroke**	
**Subgroup**	**DiD Estimate (95% CI)**	**Pre-trend P-value**	**DiD Estimate (95% CI)**	**Pre-trend P-value**
By acquisition cohort				
2012	-0·019 (-0·033 to -0·004)	0·09	0·001 (-0·015 to 0·018)	0·28
2013	-0·019 (-0·032 to -0·005)	0·29	-0·003 (-0·018 to 0·013)	0·24
2014	-0·019 (-0·033 to -0·005)	0·44	-0·013 (-0·029 to 0·003)	0·08
2015	-0·019 (-0·035 to -0·004)	0·33	-0·022 (-0·041 to -0·004)	0·16
2016	-0·001 (-0·021 to 0·020)	0·23	-0·022 (-0·045 to 0·001)	0·45
By bed utilization rate				
High utilization	-0·011 (-0·022 to -0·001)	0·54	0·000 (-0·014 to 0·014)	0·99
Low utilization	-0·014 (-0·026 to -0·001)	0·03	-0·007 (-0·020 to 0·006)	0·34
By market competition				
High competition	-0·008 (-0·021 to 0·005)	0·22	0·002 (-0·012 to 0·016)	0·84
Low competition	-0·014 (-0·023 to -0·004)	0·23	-0·007 (-0·019 to 0·005)	0·28

Supplementary Table 4- Difference-in-differences estimates of the association between hospital acquisition and all outcomes, stratified by control group system affiliation. Data represent difference-in-differences (DiD) estimates of the association between hospital acquisition and primary patient health outcomes (excess 30-day mortality, raw 30-day mortality, 30-day readmissions), staffing intensity, patient volume, and service availability. Results are stratified to compare acquired hospitals against either independent (nonsystem) control hospitals or system-affiliated control hospitals. Medical doctor (MD) and nurse intensity are calculated as the number of clinicians per 1,000 patient-days (PD) (details in Appendix B). Data in parentheses represent 95% confidence intervals.

	AMI		Stroke	
Primary outcomes	Independent controls	System-affiliated controls	Independent controls	System-affiliated controls
Excess 30-day mortality	-0·013 (-0·021 to -0·004)	-0·014 (-0·023 to -0·004)	-0·005 (-0·015 to 0·005)	-0·003 (-0·014 to 0·007)
Secondary outcomes				
Raw 30-day mortality	-0·010 (-0·020 to 0·000)	-0·011 (-0·021 to 0·000)	-0·008 (-0·018 to 0·003)	-0·006 (-0·017 to 0·006)
30-day readmission rate	0·001 (-0·011 to 0·013)	0·002 (-0·010 to 0·015)	0·000 (-0·008 to 0·008)	-0·001 (-0·010 to 0·007)
Staffing and volume				
MD intensity (per 1000 PD)	0·06 (-0·01 to 0·14)	0·03 (-0·06 to 0·12)	-0·05 (-0·14 to 0·03)	-0·04 (-0·13 to 0·04)
Nurse intensity (per 1000 PD)	0·54 (-0·45 to 1·54)	1·94 (0·48 to 3·41)	-0·35 (-1·78 to 1·08)	1·61 (-0·47 to 3·69)
Patient volume	1·55 (-1·02 to 4·13)	1·03 (-2·08 to 4·13)	-2·45 (-6·04 to 1·15)	-1·85(-5·84 to 2·15)
Service availability				
Probability of operating cathlab/stroke unit	0·06 (0·00 to 0·12)	0·07 (0·01 to 0·13)	0·04 (-0·02 to 0·10)	0·06 (0·00 to 0·12)

Supplementary Table 5- Difference-in-differences estimates of spillover effects on neighboring hospitals within 8 km and 15 km of an acquired hospital for acute myocardial infarction (AMI) and stroke. Data represent difference-in-differences estimates of the association between hospital acquisition and outcomes at nonacquired hospitals located within 8-km and 15-km radii of an acquired hospital. Outcomes include patient health outcomes (excess and raw 30-day mortality; 30-day readmissions), staffing intensity, patient volume, and service availability (cardiac catheterization laboratory and stroke unit). Medical doctor (MD) and nurse intensity are calculated as the number of clinicians per 1000 patient-days (PD) (details in Appendix B). CI stands for confidence interval. The reported pre-trend P-values test the joint hypothesis that pre-transaction coefficients from the dynamic event study are zero.

	**Acute Myocardial Infarction (AMI)**		**Stroke**	
**Outcome**	**DiD Estimate (95% CI)**	**Pre-trend P-value**	**DiD Estimate (95% CI)**	**Pre-trend P-value**
Within 8-km radius				
Patient outcomes				
Excess 30-day mortality	0·003 (-0·004 to 0·011)	0·50	0·005 (-0·003 to 0·012)	0·97
Raw 30-day mortality	0·007 (-0·002 to 0·015)	0·72	0·007 (-0·001 to 0·015)	0·70
30-day readmission rate	-0·004 (-0·012 to 0·005)	0·40	0·001 (-0·007 to 0·009)	0·44
Staffing and volume				
MD intensity (per 1000 PD)	0·04 (-0·13 to 0·21)	0·22	0·14 (0·01 to 0·27)	0·21
Nurse intensity (per 1000 PD)	-0·98 (-2·77 to 0·81)	0·30	-0·70 (-2·48 to 1·08)	0·05
Patient volume (No. of cases)	-2·85 (-6·71 to 1·00)	0·70	2·85 (-1·92 to 7·61)	0·28
Service availability				
Probability of operating cathlab/stroke unit	-0·02 (-0·05 to 0·00)	0·05	-0·04 (-0·06 to -0·02)	0·04
Within 15-km radius				
Patient outcomes				
Excess 30-day mortality	0·001 (-0·004 to 0·006)	0·49	0·001 (-0·004 to 0·007)	0·62
Raw 30-day mortality	0·005 (-0·001 to 0·011)	0·76	0·003 (-0·003 to 0·008)	0·93
30-day readmission rate	-0·005 (-0·011 to 0·002)	0·04	-0·003 (-0·009 to 0·003)	0·87
Staffing and volume				
MD intensity (per 1000 PD)	0·07 (-0·02 to 0·17)	0·14	0·08 (0·00 to 0·17)	0·38
Nurse intensity (per 1000 PD)	-0·37 (-1·22 to 0·48)	0·22	-0·49 (-1·78 to 0·81)	0·06
Patient volume (No. of cases)	-0·18 (-2·91 to 2·56)	0·66	1·76 (-1·42 to 4·95)	0·10
Service availability				
Probability of operating cathlab/stroke unit	-0·02 (-0·06 to 0·01)	0·45	-0·03 (-0·06 to 0·00)	0·28
