# Supplementary material for: Hospital acquisitions and 30-day mortality after acute myocardial infarction and stroke in Germany: a quasi-experimental cohort study
Source: Lancet Reg Health Eur. 2026 Jul 23;68:101788. doi: 10.1016/j.lanepe.2026.101788 (PMC13425888; doi:10.1016/j.lanepe.2026.101788)
Supplement: Translated Abstract [file mmc2.docx]

This translation in German was submitted by the authors and we reproduce it as supplied. It has not been peer reviewed. Our editorial processes have only been applied to the original abstract in English, which should serve as reference for this manuscript.

**Krankenhausübernahmen und 30-Tage-Mortalität nach akutem Myokardinfarkt und Schlaganfall in Deutschland: eine quasi-experimentelle Kohortenstudie**

**Zusammenfassung**

**Hintergrund:** Die Konsolidierung von Krankenhäusern hat weltweit zugenommen, jedoch ist die Evidenz bezüglich der Auswirkungen auf die Outcomes weiterhin gemischt, insbesondere in universellen Gesundheitssystemen mit regulierten Preisen. Wir untersuchten, ob Krankenhausübernahmen mit Veränderungen der 30-Tage-Exzessmortalität bei Krankenhausaufnahmen wegen akutem Myokardinfarkt (AMI) und Schlaganfall assoziiert waren.

**Methoden:** In dieser Kohortenstudie wurde ein gestaffeltes Differenz-von-Differenzen-Design (*staggered difference-in-differences*) angewendet, um deutsche Krankenhäuser zu analysieren, die AMI- oder Schlaganfallversorgung anbieten (2009–2019). Wir verglichen Leistungsveränderungen drei Jahre vor und nach der Übernahme zwischen übernommenen Krankenhäusern (n=125 für AMI; n=121 für Schlaganfall) und Kontrollkrankenhäusern mit stabilen Eigentumsverhältnissen (n=821 für AMI; n=830 für Schlaganfall). Primäre Endpunkte waren die Raten der 30-Tage-Exzessmortalität. Sekundäre Endpunkte umfassten die Personaldichte im medizinischen Bereich sowie die Vorhaltung von Herzkatheterlaboren und Stroke Units (Schlaganfallstationen).

**Ergebnisse:** Eine Übernahme war innerhalb von drei Jahren mit einem Rückgang der 30-Tage-Exzessmortalität bei AMI um 1,24 Prozentpunkte (PP) assoziiert (95%-KI –2,04 bis –0,44; p=0,002). Diese Verbesserung ging mit einer Erhöhung der Wahrscheinlichkeit für die Vorhaltung von Herzkatheterlaboren um 6,63 PP einher (0,75 bis 12,52; p=0,03). Bei Schlaganfall wies die Assoziation mit der 30-Tage-Exzessmortalität auf einen klinisch relevanten, jedoch statistisch nicht signifikanten Rückgang um 0,43 PP hin (–1,38 bis 0,53; p=0,44). Die Personaldichte in der Pflege stieg im Jahr der Übernahme sowohl bei AMI als auch bei Schlaganfall vorübergehend an.

**Interpretation:** In einem regulierten Festpreissystem waren Übernahmen signifikant mit einer reduzierten AMI-Mortalität und einer erweiterten interventionellen Infrastruktur assoziiert. Die Ergebnisse deuten darauf hin, dass eine Konsolidierung die Outcomes verbessern kann, indem Kapital für lebensrettende Investitionen mobilisiert wird. Während kapitalintensive Erkrankungen wie AMI rasch profitieren, erfordern komplexe Behandlungspfade wie beim Schlaganfall wahrscheinlich zusätzliche klinische Integrationsstrategien.

**Finanzierung:** Deutsche Forschungsgemeinschaft (DFG).
